# Quantum-accessible reinforcement learning beyond strictly epochal environments

**DOI:** 10.1007/s42484-021-00049-7

**Published:** 2021-08-02

**Authors:** A. Hamann, V. Dunjko, S. Wölk

**Affiliations:** 1 Institut für Theoretische Physik, Universität Innsbruck, Technikerstraße 21a, 6020 Innsbruck, Austria; 2 LIACS, Leiden University, Niels Bohrweg 1, 2333 CA Leiden, The Netherlands; 3 Present Address: Institute of Quantum Technologies, German Aerospace Center (DLR), D-89077 Ulm, Germany

**Keywords:** Reinforcement learning, Quantum-classical hybrid agent, Amplitude amplification

## Abstract

In recent years, quantum-enhanced machine learning has emerged as a particularly fruitful application of quantum algorithms, covering aspects of supervised, unsupervised and reinforcement learning. Reinforcement learning offers numerous options of how quantum theory can be applied, and is arguably the least explored, from a quantum perspective. Here, an agent explores an environment and tries to find a behavior optimizing some figure of merit. Some of the first approaches investigated settings where this exploration can be sped-up, by considering quantum analogs of classical environments, which can then be queried in superposition. If the environments have a strict periodic structure in time (i.e. are strictly episodic), such environments can be effectively converted to conventional oracles encountered in quantum information. However, in general environments, we obtain scenarios that generalize standard oracle tasks. In this work, we consider one such generalization, where the environment is not strictly episodic, which is mapped to an oracle identification setting with a changing oracle. We analyze this case and show that standard amplitude-amplification techniques can, with minor modifications, still be applied to achieve quadratic speed-ups. In addition, we prove that an algorithm based on Grover iterations is optimal for oracle identification even if the oracle changes over time in a way that the “rewarded space” is monotonically increasing. This result constitutes one of the first generalizations of quantum-accessible reinforcement learning.

## Introduction

In the last few years, there has been much interest in combining quantum computing and machine learning algorithms. In the domain of quantum-enhanced machine learning, the objective is to utilize quantum effects to speed-up or otherwise enhance the learning performance. The possibilities for this are numerous (Dunjko and Briegel 2018). E.g. variational circuits can be used as a type of “quantum neural network” (more precisely, using them as function approximators which cannot be evaluated efficiently on a conventional computer), which can be trained as a supervised learning (classification) (Havlícek et al. 2019; Farhi and Neven 2018) or unsupervised learning model (generative models) (Aimeur et al. 2013). There also exist various approaches where algorithmic bottlenecks of classical algorithms are sped-up, via annealing methods (Farhi and Neven 2018), quantum linear-algebraic methods (Harrow et al. 2009), or via sampling enhancements (Dunjko et al. 2016). If the data is assumed to be accessible in a quantum form (“quantum database”), then anything from polynomial to exponential speed-ups of classical algorithms may be possible (Biamonte et al. 2017; Dunjko and Briegel 2018; Chia et al. 2019; Gyurik et al. 2020)^1^.

Modern reinforcement learning (RL), an interactive mode of learning, combines aspects of supervised and unsupervised learning, and consequently allows a broad spectrum of possibilities how quantum effects could help.

In RL (Sutton and Barto 1998; Russell and Norvig 2003; Briegel and De las Cuevas 2012), we talk about a learning agent which interacts with an environment, by performing actions, and perceiving the environmental states, and has to learn a “correct behavior”—the optimal policy—by means of a feedback rewarding signal. Unlike a stationary database, the environment has its own internal memory (a state), which the agent alters with its actions.

In quantum-enhanced RL, we can identify two basic scenarios: (i) where quantum effects can be used to speed up the internal processing (Paparo et al. 2014; Jerbi et al. 2019), and the interaction with the environment is classical, and (ii) where the interaction with the environment (and the environment itself) is quantum. The first framework for such “quantum-accessible” reinforcement learning modeled the environment as a sequence of quantum channels, acting on a communication register, and the internal environmental memory—this constitutes a direct generalization of an unknown environment as a map-with-memory (other options are discussed shortly). In this case, the action of the environment cannot be described as unitary mapping, without considering the entire memory of the environment. In general, this memory is inaccesible to the agent. However, as discussed in Dunjko et al. (2016), under the assumptions that the environmental memory can be purged or uncomputed in pre-defined periods, such blocks of interaction do become a (time-independent) unitary and amenable to oracle computation techniques. For instance, in Dunjko et al. (2016), it was shown that the task of identifying a sequence of actions which leads to a first reward (a necessary step before any true learning can commence) can be sped up using quantum search techniques, and in Dunjko et al. (2017), it was shown how certain environments encode more complex oracles—e.g. Simon’s oracle and Recursive Fourier Sampling oracles, leading to exponential speed-ups over classical methods.

For the above techniques to work, however, the purging of all of environmental memory is necessary to achieve time-independent unitary mappings. However, real task environments are typically not (strictly) episodic, motivating the question of what can be achieved in these more general cases. Here, we perform a first step towards generalization by considering environments where the length of the episode can change, but this is signaled and the estimate of the episode lengths is known. This RL scenario is well-motivated and fortunately maps to an oracle identification problem where the oracles change. While this generalizes standard oracular settings, it is still sufficiently simple such that we can employ standard techniques (essentially amplitude amplification) and prove the optimality of our strategies for oracle identification problems with changing oracles and increasing rewarded space.

The paper is organized as follows. We will first summarize the basics scenario of quantum-accessible reinforcement learning in Section 2 and discuss the mappings from constrained (episodic) RL scenarios to oracle identification. We show how this must be generalized for more involved environments, prompting our definition of the “changing oracle” problem stemming from certain classes of RL environments. In Section 3, we focus on the changing oracle problem, analyze the main regimes, and provide an upper bound for the average success probability for the case of monotonically increasing rewarded space in Section 3.1. We proof in Section 3.2 that performing consecutive Grover iterations saturates this bound. We then discuss the more general case of only overlapping rewarded spaces in Section 3.3. In Section 3.4, we provide a numerical example demonstrating the possible advantages of consecutive Grover iterations with changing oracles. We conclude by summarizing our results, by discussing possible extensions, and by noting on the implications of our results of the changing oracle problem for QRL in Section 4.

## Quantum-accessible reinforcement learning

RL can be described as an interaction of a learning agent *A* with a task environment *E* via the exchange of messages out of a discrete set which we call actions $\mathcal {A}=\lbrace a_{j}\rbrace $ (performed by the agent) and percepts $\mathcal {S}=\lbrace s_{j}\rbrace $ (issued by the environment). In addition, the environment also issues a scalar reward $\mathcal {R}=\lbrace r_{j}\rbrace $, which informs the agent about the quality of the previous actions and can be defined as being a part of the percepts. The goal of the agent is to receive as much reward as possible in the long term.

In theory of RL, the most studied environments are exactly describable by a Markov decision process (MDP). An MDP is specified by a transition mapping and a reward function . The transition mapping *T* specifies the probability of the environment transiting from state *s* to $s^{\prime }$, provided the agent performed the action *a*, whereas the reward function assigns a reward value to a given action of an agent in a given environmental state.

Note that in standard RL, the agent does not have a direct access to the mapping *T*, but rather to learn it, it must explore, i.e. to act in the environment which is governed by *T*. On the other hand, in dynamical programming problems (intimately related to RL), one often assumes access to the functions *T* and *R* directly. This distinction leads to two different tasks on how agent-environment interaction can be quantized.

In recent works (Cornelissen 2018; Neukart et al. 2018; Levit et al. 2017), coherent access to the transition mapping *T* is assumed; in this case, lower quantum bounds for finding the optimal policy have been found (Ronagh 2019).

In this paper, we consider the other class of generalization, proposed first in Dunjko et al. (2016). Here, the agent-environment interaction is modeled as a communication between an agent (A) and the environment (E) over a joint communication channel (C), thus in a three-partite Hilbert space ${\mathscr{H}}_{E}\otimes {\mathscr{H}}_{C} \otimes {\mathscr{H}}_{A} $, denoting the memory of the environment, the communication channel, and the memory of the agent. The two parties A and E interact with each other by performing alternately completely positive trace preserving (CPTP) maps on their own memory and the communication channel. Different AE combinations are defined as equivalent in the classical sense, if their interactions are equivalent under constant measurements of C in the computational basis. For classical luck favoring AE settings with a deterministic strictly epochal environment E, it is possible to create a classical equivalent quantum version A^*q*^*E*^*q*^ which outperforms AE in terms of a given figure of merit as shown in Dunjko et al. (2016).

### Strictly epochal environments

This can be achieved by slightly modifying the maps as to purge the environmental memory which couples to the overall interaction preventing a unitary time evolution of the agents memory. A detailed discussion of this procedure and necessary condition on the setting is outlined in Dunjko et al. (2016). However, for our setting, it is sufficient that the interaction of the agent with the environment can be effectively described as oracle queries. Specifically if environments are strictly episodic, meaning after some fixed number of steps the setting is re-set to an initial condition, then the environmental memory can be uncomputed or released to the agent at the end of an epoch. With this modification (called memory scavenging and hijacking in earlier works), blocks of interactions effectively act as one, time-independent unitary *O*, which can be queried using standard quantum techniques to obtain an advantage. We encode different actions the agent can perform, like *a* ∈{0,1}, into orthogonal quantum states, i.e. as {|0〉, |1〉}. As a result, the complete sequence of actions **a** = *a*_1_,⋯ ,*a*_*M*_ the agent executes during one epoch of length *M* is encoded in the product state |**a**〉 = |*a*_1_〉⊗|*a*_2_〉⊗⋯ ⊗|*a*_*M*_〉. For strictly epochal environments, it is possible to re-express the effect of the environment by an unitary oracle

1 $$ O {|\mathbf{a}\rangle} = \left\lbrace \begin{array}{cc} -{|\mathbf{a}\rangle}& \text{if} \mathbf{a} \in W \\ {|\mathbf{a}\rangle}&\text{else} \end{array}\right. $$

Here, *W* denotes the rewarded space containing all sequences of actions of length *M* which obtained a reward *r*(**a**) larger than a predefined limit.

A learning agent can use such an oraculized environment to find rewarded action sequences faster. For this purpose, the agent prepares an equal superposition state of all possible action sequences

2 $$ {|\psi\rangle}=\frac{1}{\sqrt{N}}\sum\limits_{i=1}^{N}{|\mathbf{a}_{i}\rangle} $$

with typically $N=|\mathcal {A}|^{M}$. Then, it interacts with the environment and thus effectively queries the oracle *O*. Afterwards, it performs a reflection over the initial state |*ψ*〉. In this way, it can perform amplitude amplification performing consecutive Grover iterations (Grover 1997; 1998; Brassard et al. 2000) *G*_*ψ*_|*ψ*〉 with
3The agent can increase the probability to find a first rewarded sequence by performing several rounds of amplitude amplification.

The first found rewarded action sequence is in general not the optimal one. However, in general, it provides information which can help to find an optimal strategy. A quantum-enhanced learning agent can use the found rewarded sequence of actions to learn by using classical policy updates. Thus, quantum-enhanced reinforcement learning combines quantum search and classical reinforcement learning as, e.g. demonstrated experimentally in Saggio et al. (2021). An agent with access to such an oracle thus finds in average the first rewarded sequence faster which increases in so-called luck-favoring settings (Dunjko et al. 2016) also the probability to be rewarded in the future. This approach leading to a quadratic speed-up in exploration can be applied to many settings and even super-polynomial or exponential improvements can be generated for special RL settings (Dunjko et al. 2017).

### Beyond strictly epochal environments

The simplest scenarios of task environments, which cannot be reformulated as an oracular problem, are arguably those which involve two oracles. We will consider this slight generalization in this work, as it still allows for a relatively simple treatment. This setting includes environments which simply change as a function of time such as reinforcement learning for managing power consumption or channel allocation in cellular telephone systems (Han 2018; Tesauro et al. 2008; da Silva et al. 2006; Singh and Bertsekas 1996). If the instances of change are known, again the blocking is possible, in which case we obtain the setting where we can realize access to an oracle but which changes as a function of time. Closely related to this is a more specific case of variable episode length. This setting, although more special, is in particular interest in RL. Episodic environments are usually constructed by taking an arbitrary environment, and establishing a cut-off after a certain number of steps. The resulting object is again an environment derived from the initial setting. This construction is special in that given any sequence of actions **a** which is rewarding in a derived environment with cut-off after *m* steps, any sequence of actions in the environment which has a larger cut off *M* > *m* which has **a** as a prefix is rewarded in the second. An example for such an environment is the Grid-world problem which consists in navigating a maze and the task is to find a specific location that is rewarded (Russell and Norvig 2003; Sutton and Barto 1998; Melnikov et al. 2018).

The classical scenarios described above, under oraculization techniques, map onto the changing oracle problem (described in detail in the following section) where at a given time an oracle $\tilde {O}$ is exchanged by a different oracle *O*. This generalization especially captures the scenario of a single increment of an epoch length from *m* to *M* > *m* for search in QRL. In this special case, the rewarded space $\tilde {W}$ of $\tilde {O}$ is a subspace of *W* of *O*. We will proof that the optimal algorithm in this case is given by a Grover search with a continuous coherent time evolution using both oracles consecutively. However, continuing the coherent time evolution of a Grover search can be suboptimal when $\tilde {W}\not \subset W$. The arguments following in the next section can be used iteratively to describe multiple changes/increments of the rewarded space.

## The changing oracle problem

The situation above can be abstracted as a “changing oracle” problem which we specify here. As suggested, we consider an “oracle” to be a standard phase-flip oracle, such that *O*|*x*〉 = (− 1)^*f*(*x*)^|*x*〉, where $f: X \rightarrow \{0,1\}$ is a characteristic function on a set of elements *X*, with |*X*| = *N*; in our case *X* denotes sequences of actions of some prescribed length. The rewarded set is denoted by *W* = {*x* ∈ *X*|*f*(*x*) = 1}, and the states |*x*〉 denote a (known) orthonormal basis.

In the changing oracle problem, we consider two oracles $\tilde {O}$ and *O*, with respective rewarded sets $\tilde {W}$ and *W*. The problem specifies two time intervals, phases, in which only one of the two oracles is available: time-steps 1 ≤ *k* ≤ *K* during which only access to $\tilde {O}$ is available, and time-steps *K* + 1 ≤ *k* < *K* + *J* during which only access to the second oracle *O* is available.

For simplicity, we assume that the values of *K*, *J*, *N* as well as the sizes of the rewarded sets $|\tilde {W}|=\tilde {n}$ and |*W*| = *n* are known in advance, and in general, the objective is to either output an $x \in \tilde {W}$ before *K*, or, to output *x* ∈ *W* in the remainder of the time. We will refer to both *x* as the solution. However, the exact time when the oracle changes, and does *K* and *J*, is not important and can be unknown as we show later. Unless *K* is in ${\Omega }(\sqrt {N/\tilde {n}})$, in general attempts to find a solution in the first phase will have a very low success probability no matter what we do due to the optimality of Grover’s search. However, even in this case, having access to $\tilde {O}$ in the first phase may improve our chances to succeed in the second. This is the setting we consider.

The optimal strategies vitally depend on the known relationship between *W* and $\tilde {W}$. We will first briefly discuss all possible setting before focusing on the most interesting cases. Note, in this paper, we are not looking for a strategy which uses a minimal number of queries until a solution is found, but rather, a strategy which maximizes the success probability for a fixed number of queries. However, it is also known that Grover’s search achieves the fastest increase of success probability (Zalka 1999). Note, the here described algorithms can be also used to optimize the number of queries. However, the corresponding figure of merit, which needs to be optimized, has to be defined precisely for such tasks.

In the worst case, there may be no known correlation between *W* and $\tilde {W}$. In this case, we have no advantage from having access to $\tilde {O}$, and the optimal strategy is a simple Grover’s search in the second phase.

Another case with limited interest is when *W* and $\tilde {W}$ are known to be disjointed. In this case, the first oracle might be used to constrain the search space to the complement $\tilde {W}^{c},$ which contains *W*. The lower bounds for this setting are easy to find: we can assume that at *K* the set $\tilde {W}$ is made known (any state we could have generated using $\tilde {O}$ can be generated with this information). However, in this case, the optimal strategy is still to simply apply quantum search over the restricted space $\tilde {W}^{c}$ if it can be fully specified. But since we most often encounter cases where $\tilde {n}=|\tilde {W}|$ is (very) small compared to *N*, the improvement that could be obtained is also minor.

Similar reasoning follows also when the sets are not disjoint, but the intersection is small compared not just to *N*, but to |*W*| and $|\tilde {W}|$. In this case, again we can find lower bounds by assuming that the non-overlapping complement becomes known. In addition, we assume that we can prepare any quantum state, which has an upper bound on the overlap with any state corresponding to the intersection, $x \in W \cap \tilde {W}$. Then, the optimal strategy is again governed by the optimality of Grover-based amplitude amplification 2

This brings us to the situations which are more interesting, specifically, when the overlap $W_{a}=W \cap \tilde {W}$ is large (see Appendix A for exact definition).

Due to our motivation stemming from aforementioned RL settings, we are particularly interested in the case when $\tilde {W} \subseteq {W},$ for which we give the optimal strategy, which turns out to be essentially Grover’s amplification where we “pretend” that the oracles hadn’t changed.

The other cases, ${W} \subseteq \tilde {W}$, and the more generic case where the overlap is large, but no containment hold are less interesting for our purpose, so we briefly discuss the possible strategies without proofs of optimality.

### Increasing rewarded spaces: upper bound on average final success probabilities

In the following, we consider the above-described changing oracle problem with monotonically increasing rewarded spaces $\tilde {W} \subseteq {W}$ and derive upper bounds for the maximal average success probability *p*_*K*+*J*_ of finding an element *x* ∈ *W* at the end of the second phase. The changing oracle problem is outside the standard settings for which various lower bounding techniques have been developed (Arunachalam et al. 2019; Ambainis 2002; 2006), but the setting is simple enough to be treatable by modifying and extending techniques introduced to lower bound unstructured search problems (Zalka 1999).

To find upper bounds of the success probability, we first prove that we can restrict our search for optimal strategies to averaged strategies as defined in Appendix B. This induces certain symmetries which restrict the optimization to an optimization of two angles *α* and *Δ*, one for each phase. Finally, we derive bounds *α*(*K*) and *Δ*(*J*) for these angles depending on *K*, *J* which in turn restrict the optimal success probability *p*_*K*+*J*_.

The search for an optimal strategy can be limited to strategies based on pure states and unitary time evolutions since it is possible to purify any search strategy by going from a Hilbert space ${\mathscr{H}}_{A}$ spanned by {|*x*〉} into a larger Hilbert space ${\mathscr{H}}_{AB}={\mathscr{H}}_{A}\otimes {\mathscr{H}}_{B}$. As a consequence, every search strategy *T* = ({*U*_*k*_},|*ψ*(0)〉) based on *K* + *J* oracle queries can be described by a set of *K* + *J* unitaries *U*_*k*_ and initial state |*ψ*(0)〉. Our knowledge about possible rewarded items after *k* oracles queries is then encoded in the quantum state

4 $$ {|\psi(k)\rangle}=U_{k}O_{k} {\cdots} U_{1}O_{1}{|\psi(0)\rangle} $$

with $O_{k}=\tilde {O}$ for 1 ≤ *k* ≤ *K* and *O*_*k*_ = *O* for *K* + 1 ≤ *k* ≤ *J*. The success probability at the end of the second phase is then given by

5 $$ p_{K+J}=\text{Tr }[P_{\mathcal{W}}{|\psi(K+J)\rangle}{\langle\psi(K+J)|}] $$

with
6Our goal is to maximize the success probability *p*_*K*+*J*_ average over all possible functions $\tilde {f}(x)$ and *f*(*x*) with fixed sizes of the rewarded spaces $|\tilde {W}|=\tilde {n}$ and $|{W}|={n}\geq \tilde {n}$. Different realization of $\tilde {f}(x)$ and *f*(*x*) can be generated by substituting all oracle queries *O*_*k*_ by *σO*_*k*_*σ*^‡^ and the projector $P_{\mathcal {W}}$ by $\sigma P_{\mathcal {W}}\sigma ^{\dagger }$ where *σ* denote a permutation operator acting on ${\mathscr{H}}_{A}$. As a consequence, an optimal strategy is a strategy T which maximizes

7 $$ \bar{p}_{T}=\frac{1}{N!}\sum\limits_{\sigma \in {\Sigma}_{A}}p_{T}(\sigma) $$

with

8 $$ \begin{array}{@{}rcl@{}} p_{T}(\sigma)&=&\text{Tr }\left[\sigma P_{\mathcal{W}}\sigma^{\dagger} {|\psi(k,\sigma)\rangle}{\langle\psi(k,\sigma)|}\right] \end{array} $$

9 $$ \begin{array}{@{}rcl@{}} {|\psi(k,\sigma)\rangle}_{AB}&=&U_{k}\sigma O_{k}\sigma^{\dagger}{\cdots} U_{1} \sigma O_{1}\sigma^{\dagger} {|\psi(0)\rangle}_{AB}\end{array} $$

at the end of the second phase such that *k* = *K* + *J*. Here, Σ_*A*_ denotes the set of all possible permutations in ${\mathscr{H}}_{A}$.

We can further limit the search for optimal strategies to averaged strategies $\bar {T}$ as defined Appendix B because

#### **Lemma 1**

The success probability $p_{\bar {T}}(\sigma )$ of the averaged strategy $\bar {T}$ is equal to the average success probability $\bar {p}_{T}$ of the strategy *T* for every permutation *σ* ∈Σ_*A*_.

as proven in Appendix B. In the following, we consider only average strategies such that $p=\bar {p}$ and therefore omit the “bar” denoting an average value.

In addition, these strategies lead to symmetry properties of the unitaries *U*_*k*_ and resulting states |*ψ*(*k*)〉 under permutations *σ* as outlined in detail in Appendix B. Therefore, we can restrict the initial states |*ψ*(0)〉 to states with equal probability $q(x)=\text {Tr }[({|\psi \rangle }_{AB}{\langle \psi |})\cdot \left ({|x\rangle }_{A}{\langle x|}\otimes \mathbf {1}_{B}\right )]$ for all elements *x*. An example for such a symmetric state is the initial state of the Grover search algorithm given by the equal superposition ${\sum }_{x}{|x\rangle }_{A}/\sqrt {N}$. Yet, many other symmetric initial states are possible due to the additional degrees of freedom resulting from the additional Hilbert space ${\mathscr{H}}_{B}$. The unitaries *U*_*k*_ cannot break the symmetry between elements |*x*〉. Only the oracles $\tilde {O}$ and *O* can break the symmetry between rewarded and not-rewarded elements.

These symmetry properties will limit the optimization overall strategies to an optimization of a few parameters or angles as we will outline below. These parameters are then again upper bounded by the optimality of Grover search.

We can decompose the state |*ψ*(*K*)〉 at the end of the first phase into a rewarded and a not-rewarded component with respect to the eigenstates of the second oracle *O*. The not-rewarded or losing component |*ℓ*〉 = |*ℓ*_*s*_〉 is symmetric with respect to elements $x\in {\mathscr{L}}$. However, the rewarded component |*w*〉 is not completely symmetric because $\tilde {O}$ breaks the symmetry between elements $x\in \tilde {W}\cap W$ and $x\in W\setminus \tilde {W}$. Thus, we can further decompose the rewarded component into a symmetric component |*w*_*s*_〉 and a component |*w*_⊥_〉 orthogonal to it (see Appendix C). As a result, the state |*ψ*(*K*)〉 is given by

10 $$ {|\psi(K)\rangle}= \cos \varepsilon {|\phi_{s}\rangle}+ \sin \varepsilon {|\phi_{\perp} \rangle}  $$

with the symmetric component

11 $$ {|\phi_{s}\rangle}=\sin \phi {|w_{s}\rangle}+\cos \phi {|\ell_{s}\rangle}\\ $$

and the orthogonal rewarded component

12 $$ {|\phi_{\perp} \rangle}={|w_{\perp} \rangle}. $$

The angles *ε* and *ϕ* are parameters depending on the strategy performed during the first phase. Their values are bounded by the success probability at the end of the first phase given by

13 $$ p_{K}=\cos^{2}\varepsilon\sin^{2}\phi+\sin^{2}\varepsilon.  $$

The time evolution during the second phase described by *V* = *U*_*K*+*J*_*O*⋯*U*_*K*+ 1_*O* is also symmetric and thus transforms the symmetric component |*ϕ*_*s*_〉 into a symmetric component and |*w*_⊥_〉 into a component orthogonal to *V* |*ϕ*_*s*_〉. As a consequence, the final success probability *p*_*K*+*J*_ can be divided into

14 $$ p_{K+J}=\cos^{2} (\varepsilon) p_{s}+ \sin^{2}(\varepsilon) p_{\perp} $$

with (see Appendix C)

15 $$ \begin{array}{@{}rcl@{}} p_{s}&=&\text{Tr }\left[P_{\mathcal{W}}V{|\phi_{s}\rangle}{\langle\phi_{s}|}V^{\dagger} \right] \end{array} $$

16 $$ \begin{array}{@{}rcl@{}} p_{\perp}&=&\text{Tr }\left[P_{\mathcal{W}}V{|w_{\perp}\rangle}{\langle w_{\perp}|}V^{\dagger} \right]. \end{array} $$

The reward probability *p*_⊥_ of the orthogonal part is maximal if *p*_⊥_ = 1 which can be achieved if, e.g. *V* acts on |*w*_⊥_〉 as identity. We parametrize the reward probability of the symmetric part via $ p_{s}=\sin \limits ^{2}(\phi +{{{\varDelta }}}), $ where the parameter *Δ* quantifies the increase of *p*_*s*_ during the second phase. Thus, *Δ* depends on the strategy performed during the second phase. We can quantify the final success probability via

17 $$ \begin{array}{@{}rcl@{}} p_{K+J}&\leq&\cos^{2} (\varepsilon) \sin^{2}(\phi+{{{\varDelta}}})+\sin^{2}(\varepsilon) \end{array} $$

18 $$ \begin{array}{@{}rcl@{}} &\leq& 1-\cos^{2}(\varepsilon)\cos^{2}\left( \phi+{{{\varDelta}}}\right). \end{array} $$

With the help of Eq. 13, we can rewrite $\cos \limits ^{2} \varepsilon $ via $ \cos \limits ^{2}\varepsilon =(1-p_{K})/\cos \limits ^{2} \phi $ leading to

19 $$ p_{K+J}\leq 1-(1-p_{K})\frac{\cos^{2}(\phi+{{{\varDelta}}})}{\cos^{2} \phi}. $$

As a consequence, *p*_*K*+*J*_ is monotonically increasing with *p*_*K*_,*ϕ*, *Δ* provided 0 ≤ *ϕ* ≤ *π*/2 and 0 ≤ *ϕ* + *Δ* ≤ *π*/2. Thus, an optimal strategy optimizes *p*_*K*_ and *ϕ* during the first phase and *Δ* during the second phase.

If we denote by

20 $$ \sin^{2}\alpha = \text{Tr }[P_{\tilde{W}}{|\psi(K)\rangle}{\langle\psi(K)|}] $$

the reward probability at the end of the first phase according to the first oracle $\tilde {O}$, then the success probability according to the second oracle *O* at this point is given by

21 $$ p_{K}=\sin^{2}\alpha +\cos^{2}\alpha \frac{n_+}{n_++n_{\ell}} $$

following Eqs. 79 and 80 in Appendix C. Here, $n_{+}=|\mathcal {W}_{+}|$ with $\mathcal {W}_{+}=\tilde {{\mathscr{L}}}\cap W$ denotes the number of items *x* marked only by the second oracle *O* as rewarded and $n_{\ell }=|\mathcal {L|}$ the number of losing items according to *O*. Thus, *p*_*K*_ increases monotonically with *α* for 0 ≤ *α* ≤ *π*/2.

The angle *ϕ* is also upper bounded by *α* via (see Appendix C, Eq. 93)

22 $$ \tan \phi \leq \tan \alpha \sqrt{\frac{\tilde{n}(n_++n_{\ell})}{(\tilde{n}+n_+)n_{\ell}}}+\sqrt{\frac{n_+^{2}}{(\tilde{n}+n_+)n_{\ell}}}.  $$

This bound also increases monotonically with *α* for 0 ≤ *α* ≤ *π*/2. As a result, the final success probability is upper bounded by the maximal achievable angles *α* (defined via the strategy during the first phase) and *Δ* (during the second phase) within the range 0 ≤ *α* ≤ *π*/2 and 0 ≤ *ϕ*(*α*) + *Δ* ≤ *π*/2.

The angles *α* and *Δ* can be upper bound with the help of a generalization of the optimality proof of Grover’s algorithm from Zalka (1999) which can be stated in the following way

#### **Lemma 2**

Given an oracle *O* which marks exactly *n* out of *N* items as rewarded, then performing Grover’s quantum search algorithm gives the maximal possible average success probability $p_{K}=\sin \limits ^{2}[(2K+1)\nu ]$ for up to 0 < *K* < *π*/(4*ν*) − 1/2 with $\sin \limits ^{2} \nu =n/N$.

The proof of this lemma follows the optimality proof from Zalka for *n* = 1 given in Zalka (1999). We outline the difference in the proof for *n* > 1 in Appendix E. In general, the angle 2*Kν* does not only limit the maximal success probability via $p\leq \sin \limits ^{2}[(2k+1)\nu ]$ when starting from a random guess, equal to $p_{0}=\sin \limits ^{2} \nu =n/N$, but to $p\leq \sin \limits ^{2}[2k\nu +\phi ]$ when starting from any fixed initial success probability $p_{0}=\sin \limits ^{2}\phi $, as we also outline in Appendix E.

As a consequence, the maximal angle *α* is bounded by $\alpha \leq (2K+1)\tilde {\nu }$ with $\sin \limits ^{2}\tilde {\nu }=\tilde {n}/N$ which follows directly from Lemma 2 provided $(2K+1)\tilde {\nu }\leq \pi /2$. And the reward probability of *p*_*s*_ is limited by $\sin \limits ^{2}(\phi +{{{\varDelta }}})$ with *Δ* < 2*Jν* provided 2*Jν* + *ϕ* ≤ *π*/2.

### Grover search is optimal for monotonically increasing rewarded spaces

In this section, we determine the (average) success probability *p*_*K*+*J*_ for the here defined changing oracle problem obtained via a generalized Grover algorithm and show that it saturates the in Section 3.1 derived bound. Grover’s algorithm starts in a equal superposition state given by

23 $$ \begin{array}{@{}rcl@{}} {|\psi(0)\rangle}&=&\frac{1}{\sqrt{N}}\sum\limits_{x=1}^{N} {|x\rangle} \end{array} $$

24 $$ \begin{array}{@{}rcl@{}} &=&\sin\tilde{v} {|\tilde{w}\rangle}+\cos\tilde{v} {|\tilde{\ell}\rangle} \end{array} $$

with

25 $$ \begin{array}{@{}rcl@{}} {|\tilde{w}\rangle}&=&\frac{1}{\sqrt{\tilde{n}}}\sum\limits_{x\in \tilde{\mathcal{W}}} {|x\rangle} \end{array} $$

26 $$ \begin{array}{@{}rcl@{}} {|\tilde{\ell}\rangle}&=&\frac{1}{\sqrt{N-\tilde{n}}}\sum\limits_{x\in \tilde{\mathcal{L}}} {|x\rangle}. \end{array} $$

**Fig. 1:**
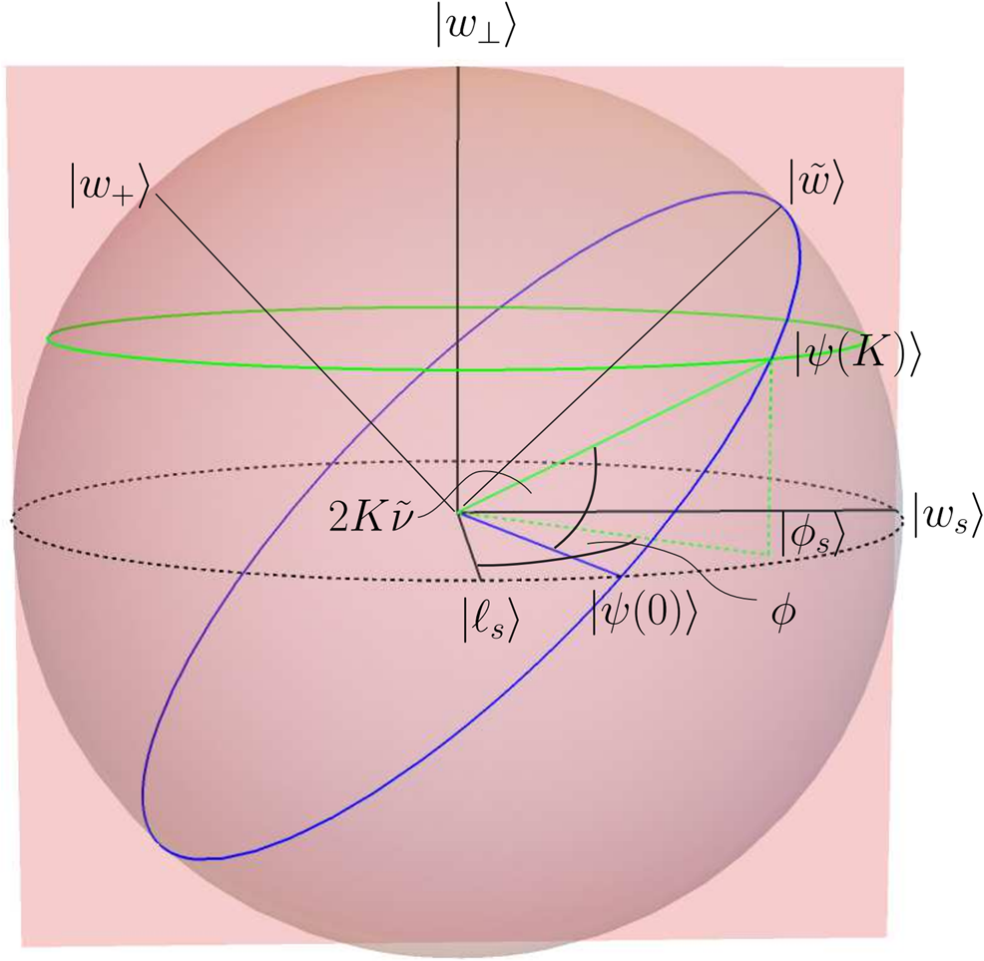
Visualization of the time evolution of |*ψ*(0)〉 under Grover iterations with changing oracles $O_{k}=\tilde {O}$ for 1 ≤ *k* ≤ *K* and *O*_*k*_ = *O* for *K* + 1 ≤ *k* ≤ *J* and $\tilde {\mathcal {W}}\subseteq \mathcal {W}$. The rewarded space (red plane) of *O* is spanned by {|*w*_*s*_〉,|*w*_⊥_〉}. The equal superposition state |*ψ*(0)〉 is rotated along the blue circle by an angle $2K\tilde {\nu }$ during the first phase leading to the state |*ψ*(*K*)〉. Consecutively, this state is rotated along the green circle changing only its component |*ϕ*_*s*_〉 but not |*w*_⊥_〉

All unitaries *U*_*k*_ for 1 ≤ *k* ≤ *K* + *J* are given by
27The time evolution during the first phase with oracle $\tilde {O}$ leads to a rotation of |*ψ*(0)〉 by an angle $2K\tilde {\nu }$ in the plane spanned by ${|\tilde {w}\rangle }$ and |*ψ*(0)〉 as depicted in Fig. 1. The state at the end of the first phase is given by

28 $$ {|\psi(K)\rangle}=\sin[(2K+1)\tilde{\nu}]{|\tilde{w}\rangle}+\cos[(2K+1)\tilde{\nu}]{|\tilde{\ell}\rangle} $$

and thus saturates the upper limit $\alpha =(2K+1)\tilde {\nu }$ leading to a maximal *p*_*K*_ and *α*. To describe the time evolution during the second phase, we perform a basis transformation into the new basis

29 $$ \begin{array}{@{}rcl@{}} {|\ell_{s}\rangle}&=&\frac{1}{\sqrt{n_{\ell}}}\sum\limits_{x\in \mathcal{L}}{|x\rangle} \end{array} $$

30 $$ \begin{array}{@{}rcl@{}} {|w_{s}\rangle}&=&\frac{1}{\sqrt{n}}\sum\limits_{x\in \mathcal{W}}{|x\rangle} \end{array} $$

31 $$ \begin{array}{@{}rcl@{}} {|w_{\perp}\rangle}&=&\sqrt{\frac{n_{+}}{\tilde{n}+n_{+}}}\sum\limits_{x\in \tilde{\mathcal{W}}} {|x\rangle}-\sqrt{\frac{n}{\tilde{n}+n_{+}}}\sum\limits_{x\in \mathcal{W}_{+}} {|x\rangle} \end{array} $$

with $\mathcal {W}_{+}=\tilde {{\mathscr{L}}}\cap \mathcal {W}$ and *n*_+_ = |*W*_+_|. The states |*w*_*s*_〉 and |*ℓ*_*s*_〉 are symmetric under permutations permuting only rewarded states with rewarded states and losing states with losing states similar to the symmetry properties of averaged strategies discussed in Appendix C. The state |*ψ*(*K*)〉 is given in this new basis by

32 $$ {|\psi(k)\rangle}=\cos \varepsilon \left( \sin \phi {|w_{s}\rangle}+\cos {|\phi\rangle} {|\ell_{s}\rangle}\right)+\sin \varepsilon {|w_{\perp} \rangle}  $$

with the angle *ϕ* defined via

33 $$ \begin{array}{@{}rcl@{}} \tan\phi &=& \frac{{\langle w_{s}|\psi(K)\rangle}}{{\langle \ell_{s}|\psi(k)\rangle}} \end{array} $$

34 $$ \begin{array}{@{}rcl@{}} &=&\tan \alpha \sqrt{\frac{\tilde{n}(n_{+}+n_{\ell})}{(\tilde{n}+n_{+})n_{\ell}}}+\sqrt{\frac{n_{+}^{2}}{(\tilde{n}+n_{+})n_{\ell}}} \end{array} $$

saturating (93). The angle *ε* is given by

35 $$ \sin \varepsilon =\sqrt{\frac{n_+}{n_++\tilde{n}}}\left[\sin(\alpha)-\sqrt{\frac{\tilde{n}}{n_++n_{\ell}}}\cos(\alpha)\right]. $$

The time evolution during the second phase, given by oracle *O* and *U*_*k*_ as given in Eq. 27, leads to a rotation of |*ψ*(*K*)〉 by an angle 2*Jν* in a plane parallel to the one spanned by |*ψ*(0)〉 and |*w*〉 as depicted in Fig. 1. As a consequence, the final state is given by

36 $$ \begin{array}{@{}rcl@{}} {|\psi(K+J)\rangle}&=&\cos \varepsilon\left[\sin(\phi+2J\nu){|w_{s}\rangle}+\cos(\phi+2J\nu){|\ell_{s}\rangle}\right]\\ &+&(-1)^J\sin \varepsilon {|w_{\perp} \rangle} \end{array} $$

leading to the maximal possible angle *Δ* = 2*Jν* and maximal $p_{\perp }=\sin \limits ^{2}\varepsilon $ and thus to the maximal possible (average) success probability *p*_*K*+*J*_.

As a result, performing consecutive Grover iterations in the first and second phase with in total *K* + *J* oracle queries leads to the maximal possible average success probability *p*_*K*+*J*_ provided $\alpha =(2K+1)\tilde {\nu }\leq \pi /2$ and *ϕ*(*α*) + 2*Jν* ≤ *π*/2.

If more queries are available such that $(2K+1)\tilde {\nu }> \pi /2$ or *ϕ* + 2*Jν* > *π*/2, then it is possible to over-rotate the state |*ψ*〉 such that applying $\tilde {O}$ or *O* less often or performing another algorithm like, e.g. fixed-point search (Yoder et al. 2014) leads to a higher success probability.

In general, the change of |*ψ*(*k*)〉 which can be created with a single oracle query *O* ($\tilde {O}$) is limited by $|{\langle \psi (k+1)|\psi (k)\rangle }|\geq \cos \limits 2\nu $ (${\cos \limits } 2\tilde {\nu }$). The maximal possible difference between |*ψ*(0)〉 and |*ψ*(*K* + *J*)〉 achievable under this constrains would require that all states |*ψ*(*k*)〉 ly within a single plane (see discussion in Zalka (1999)). However, changing the oracle in Grover’s algorithm leads to a change or tilt of the rotation plane/ axis as visualized in Fig. 1. Nevertheless, performing Grover iterations is the optimal strategy as we have proven. In addition, changing the oracle creates a component |*ϕ*_⊥_〉 which stays invariant under consecutive Grover iterations with the new oracle. Luckily, this component contains only rewarded items such that it does not prevent us from further increasing the success probability with Grover iterations if $\mathcal {\tilde {W}}\subseteq \mathcal {W}$. As a consequence, the optimality of Grover’s algorithm in the case of a changing oracle might be not surprising but is also not obvious. Especially because performing Grover’s algorithm with the maximal number of available oracle queries is not necessarily optimal if $\tilde {\mathcal {W}}$ and $\mathcal {W}$ only share a large overlap but $\tilde {\mathcal {W}}\not \subseteq \mathcal {W}$.

### Grover iterations for $\tilde {\mathcal {W}}\not \subseteq \mathcal {W}$

In the following, we investigate the performance of Grover’s algorithm if $\tilde {\mathcal {W}}$ and $\mathcal {W} $ share a large overlap (see Appendix C) but $\tilde {\mathcal {W}}\not \subseteq \mathcal {W}$. We will show that performing the maximal number *K* of oracle queries during the first phase is not always optimal depending on the number of available queries *J* in the second phase.

If $\tilde {\mathcal {W}}\not \subset \mathcal {W}$, then the perpendicular component |*ϕ*_⊥_〉, Eq. 10 also includes a losing component |*ℓ*_⊥_〉 such that the state |*ψ*(*K*)〉 can be written via

37 $$ \begin{array}{@{}rcl@{}} {|\psi(K)\rangle}&=& \cos \varepsilon {|\phi_{s}\rangle}+ \sin \varepsilon {|\phi_{\perp}\rangle} \end{array} $$

38 $$ \begin{array}{@{}rcl@{}} {|\phi_{s}\rangle}&=&\sin \phi {|w_{s}\rangle}+\cos \phi {|\ell_{s}\rangle} \end{array} $$

39 $$ \begin{array}{@{}rcl@{}} {|\phi_{\perp}\rangle}&=&\sin \chi {|w_{\perp}\rangle}+\cos \chi {|\ell_{\perp}\rangle}. \end{array} $$

**Fig. 2:**
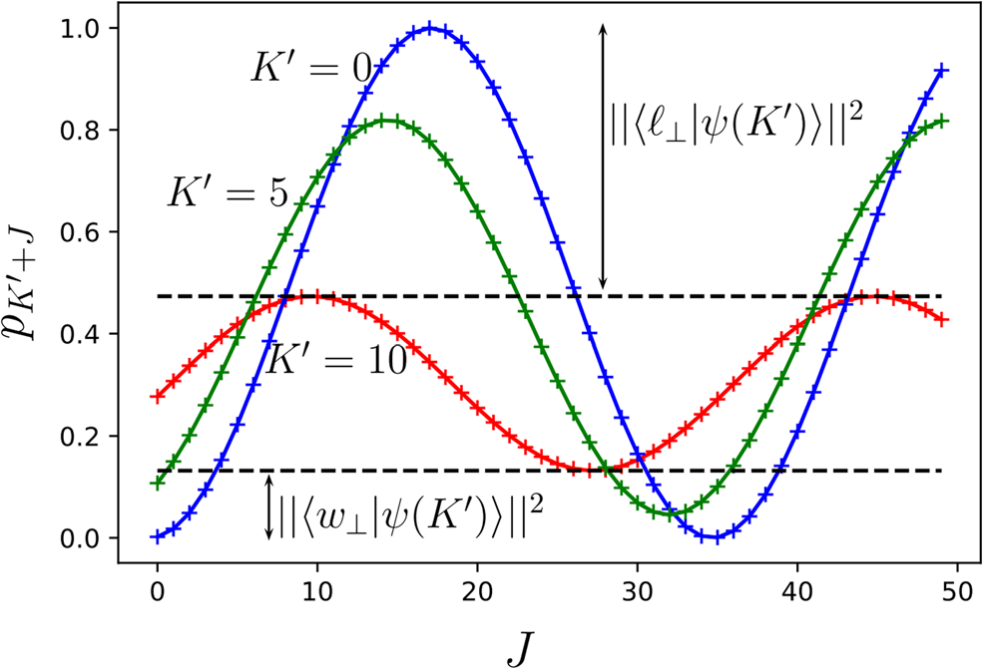
Comparison of the success probabilities $p_{K^{\prime }+J}$ for different numbers $K^{\prime },J$ of Grover iterations during the first and second phase with $K^{\prime }=0$ (blue), $K^{\prime }=5$ (green) and $K^{\prime }=10$ (red) for $n_{\ell }=5000, \tilde {n}=15, n=10,n_{+}=5$ and thus $n_{-}=10=|\mathcal {\tilde {W}}\cap {\mathscr{L}}|$

Applying Grover iterations with unitaries *U*_*k*_ as defined in Eq. 27 does not change the success probability of the component |*ϕ*_⊥_〉. It only changed the success probability of the component |*ϕ*_*s*_〉 leading to

40 $$ \begin{array}{@{}rcl@{}} p_{K+J}&=&\cos^{2}\varepsilon \sin^{2}(\phi+{{{\varDelta}}})+\sin^{2}\varepsilon \sin^{2}\chi \end{array} $$

41 $$ \begin{array}{@{}rcl@{}} &\leq&1-\sin^{2}\varepsilon\cos^{2}\chi \end{array} $$

with *Δ* = 2*Jν*. As a consequence, the success probability at the end of second phase is limited by 1 −|〈*ℓ*_⊥_|*ψ*(*K*)〉|^2^ and thus by the weight of the orthogonal losing component created during the first phase. The contribution of this component is increasing with *K* for $K<\frac {\pi }{4\tilde {\nu }}-1/2$ as shown in Fig. 2.

In this case, the success probability *p*_*K*+*J*_ is still monotonically increasing with *Δ*. Therefore, performing the maximal possible number (*J*) of Grover iterations during the second phase is still a good idea provided *ϕ* + 2*Jν* ≤ *π*/2. However, performing the maximal number (*K*) of Grover iterations during the first phase is not optimal if it leads to phases *ϕ* = *ϕ*(*K*) and *χ* = *χ*(*K*) such that

42 $$ \sin^{2} \chi(K)< \sin^{2}[2J\nu+\phi(K)]. $$

In this situation, performing less Grover iterations $K^{\prime }<K$ during the first phase can lead to a higher final success probability $p_{K^{\prime }+J}>p_{K+J}$ as shown in Fig. 2. Here, performing only $K^{\prime }=5$ instead of $K^{\prime }=10$ Grover iterations leads to higher final success probabilities for 6 ≤ *J* ≤ 11. In general, it is optimal to perform the maximal number *K* of Grover iterations during the first phase if *J* = 0 (provided $(2K+1)\tilde {\nu }<\pi /2$). However, less and less effective queries to the first oracle $\tilde {O}$ should be used the more queries to the second oracle are available as demonstrated in Fig. 2.

### Minimizing the cost to find a rewarded item

In the previous section, we have proven that it is possible to pursue a Grover-based search strategy and maintaining optimality even when the oracle is changing provided the rewarded space is monotonically increasing. We annotate that changing oracle settings have not been considered in literature before from the present perspective. Hence, it was unclear whether continuing Grover iterations in settings which changing oracles would lead to suboptimal results (compare Section 3.3). Note that the standard results for Grover search (Grover 1997; 1998; Brassard et al. 2000) can be only applied to the changing oracle problem if the two phases are treated separately. That is, a Grover search is performed during the first phase. At the end of this phase, a measurement is performed. If no rewarded item is found, another Grover search is started with queries to the second oracle.

In the following, we discuss a numerical example demonstrating the possible improvements we can gain by proceeding with a coherent Grover-based strategy in a setting where the oracle changes rather than stopping it and starting a new one.

In our example, we assume that *K* Grover iterations $\tilde {G}_{\psi }$ with $\tilde {O}$ have been performed during a first phase. Then, the oracle is changed to *O* with $\tilde {W}\subseteq W$ and an arbitrary number of queries to the second oracle are allowed. We compare in our example the following two procedures: (a) a measurement is performed immediately after the change of the oracle and (b) the coherent time evolution is continued with the new oracle before performing a measurement. In both cases, we continue with a standard Grover search based solely on the second oracle *O* if the first measurement did not reveal a rewarded item.

In many scenarios, the goal is to minimize a given cost function, such as the number of oracle queries, rather than optimizing the reward probability. Therefore, we compare in this section the cost to find a rewarded item if we either proceed or interrupt the coherent search when the oracle changes. Here, we us *C*(*j*) = 2*j* + 1 as a typical cost function for a Grover algorithm with *j* steps (see e.g. Dunjko et al. (2016) and Sriarunothai et al. (2019)).

**Fig. 3:**
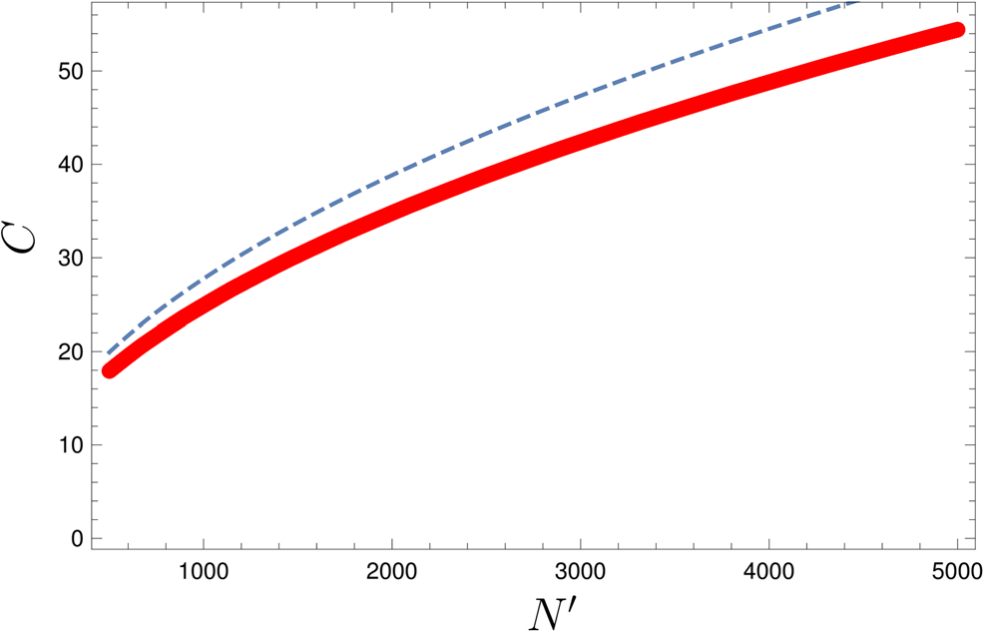
Minimal expected average cost *C* of a continuous Grover algorithm with changing oracle with optimized *j* (red) for $\psi =\sqrt {|W|/N}=1/\sqrt {N^{\prime }}$ and fixed *ϕ*_*K*_ = 0.6 and *ε*_*K*_ ≈ 0.32. The expected average cost for an interrupted Grover algorithm with fixed oracles is denoted by a blue dashed line for comparison

In the following, we neglect the cost produced by queries to the first oracle $\tilde {O}$ because this cost is identical for both cases and optimize the number of Grover iterations during the second phase (see Appendix F). In general, it is advantageous to continue the coherent time evolution if

43 $$ 4\nu\leq \pi-\arcsin\left( \frac{1}{1.38(1-\sin^{2}\varepsilon_{K})}-2\phi_{K}\right) $$

with $\sin \limits ^{2}\nu =|W|/N=1/N^{\prime }$. Here, *ϕ*_*K*_ quantifies the reward probability of the symmetric part after the first phase (compare Eq. 35) and *ε*_*K*_ the ratio between the symmetric component and the orthognal component (compare (33)). The cost functions for both procedures scale with $\sqrt {N^{\prime }}$ just like in typical amplitude amplification algorithms. The cost difference between stopping or continuing Grover’s algorithm also scales with $\sqrt {N^{\prime }}$ for fixed angles *ϕ*_*k*_ and *ε*_*k*_ as we have evaluate in Appendix F and visualized in Fig. 3.

## Conclusion

Research in quantum enhanced reinforcement learning has motivated quantum computation scenarios involving two systems, the agent and its environment, with restricted access to each other. In special cases, the interaction of the agent with its environment can be reduced to unitary oracle queries. However, general settings do not allow such a treatment due to memory effects induced by the environment.

In this paper, we generalized the basic case, where the environment acts effectively as a single fixed oracle, to settings where the oracle changes in time. This was motivated by standard grid-world type problems, where the number of consecutive actions within a single epoch can grow or shrink. We have demonstrated that the search for a rewarded action sequence of increasing length can be described as a search in a data based with fixed sequence length (equal to the maximal sequence length) but changing oracle leading to an increase of the rewarded space. We analyzed this setting and identified Grover-type amplitude amplification as optimal strategy for monotonically increasing rewarded spaces.

However, continuing coherent Grover iterations when the target space decreases will partially trap the resulting state within the losing subspace. As a consequence, the reward probability will be limited, with a limit clearly below unity, if we continue with Grover iterations after the oracle has changed.

It is easy to conceive a cascade of ever more general problems. For example, in slightly more general settings the agent might be allowed to chose if and when to change the effective oracle. In this way, the agent might combine breadth-first and depth-first search in a single coherent search for RL. Often, shorter rewarded action sequences are preferred but longer rewarded action sequences are more likely. Increasing the sequence length during a coherent quantum search will amplify the probability for shorter rewarded sequences more than for longer sequences. Combing different oracles, corresponding to different sequence length, within a single Grover search might therefore help to balanced the tradeoff between the desire for short rewarded sequences on the one side and high reward probabilities on the other. Further research investigating these possible benefits is needed. Quantum-enhanced reinforcement learning agent using different oracles might be developed for many different scenarios such as grid world, navigation or routing. In addition, many problems where we are searching for the shortest correct sequence of actions to achieve a goal, like optimal elements to create entangled photons (Melnikov et al. 2018), are equivalent to the grid-world problem.

We envision that the results presented here may become even more important in the future when large-scale quantum networks (Kimble 2008; Cacciapuoti et al. 2020) become available where the interactions is naturally quantum.

The goal in RL is in general to minimize a given cost function *C* instead of maximizes solely the success probability. In general, performing consecutive Grover iterations can be also used to minimize the average number of oracle queries necessary until a rewarded item is found. In general, we expect for our algorithm a quadratic improvement of the cost $C_{g}\propto \sqrt {C_{\text {class}}}$ compared to the cost *C*_class_ of a classical algorithm. In Section 3.4, we discussed a cost function depending solely on the number of oracle queries. We compared quantum algorithms based on a single oracle or multiple oracles leading to the costs *C*_*s*_ and *C*_*g*_, respectively. Here, we found that the difference *C*_*s*_ − *C*_*g*_ ∝ *C*_*s*_ scales with the cost.

An optimal algorithm will depend on the exact cost function we want to minimize. For example, the search algorithm described in Boyer et al. (1998) is only optimal in terms of oracle queries. However, the number of elementary qubit gates necessary to perform a Grover search can be reduced by using a recursive Grover search (Arunachalam and de Wolf 2015) which separates the database into several subgroups. In RL, queries to different oracles might be connected to different cost. In such setting, an optimal algorithm might use different oracles in a recursive way for a quantum search. In this way, improvements in terms of cost might go beyond the quadratic improvements achievable in quantum exploration.

Finally, possibly the most interesting extensions would avoid reductions of environments to unitary oracles, and identify new schemes to obtain improvements in settings which may be more applicable in real-world RL settings. We leave these more general considerations for follow-up investigations.

## Appendix A: Large overlap of $\tilde {\mathcal {W}}$ and $\mathcal {W}$

We say that the rewarded spaces $\tilde {\mathcal {W}}$ and $\mathcal {W}$ have a large overlap if increasing the probability $\tilde {p}$ for $x\in \tilde {\mathcal {W}}$ uniformly also increases the probability *p* to find $x\in {\mathcal {W}}$.

In general, optimal search strategies can be always constructed in such a way that the probability for all rewarded states *p*(*x*|*f*(*x*) = 1) are equal as outlined in Appendix B. The same holds for losing states |*x*〉 with *f*(*x*) = 0. Let $n_{a}=|\tilde {\mathcal {W}}\cap \mathcal {W}|$ (*n*_*ℓ*_) be the number of states which are marked as rewarded (not-rewarded/losing) by both oracles and $n_{-}=|\tilde {\mathcal {W}}\cap {\mathscr{L}}|$ (*n*_+_) the number of states which are rewarded only according to the first (second) oracle. Thus, the total number of items is given by *N* = *n*_*a*_ + *n*_*ℓ*_ + *n*_−_ + *n*_+_. We denote the probabilities to find any state which is always rewarded, always not rewarded, rewarded only in the first phase and rewarded only during the second phase by *p*_*a*_,*p*_*ℓ*_,*p*_−_,*p*_+_. Increasing the initial probability $\tilde {p}=p_{a}+p_{-}=(n_{a}+n_{-})/N$ during the first phase in a symmetric way as outlined in Appendix B by a factor *α* leads to

44 $$ p_a\rightarrow \alpha p_a,\quad p_-\rightarrow \alpha p_-,\quad p_{\ell}\rightarrow \beta p_{\ell},\quad p_+\rightarrow \beta p_+ $$

with

45 $$ \beta= \frac{N-\alpha (n_a+n_-)}{n_{\ell}+n_+} $$

due to normalization. This leads to a change of *p* given by

46 $$ p=p_a+p_+\rightarrow \frac{n_+}{n_{\ell}+n_+}+\alpha \frac{n_an_{\ell}-n_+n_-}{n_{\ell}+n_+}. $$

As a result, we can increase *p* by increasing $\tilde {p}$ in a symmetric way whenever

47 $$ n_an_{\ell}>n_+n_-. $$

As a result, we say $\tilde {W}$ and *W* share a large overlap if they fulfill (47).

## Appendix B: Averaged search strategies

In the following we consider search problems defined via some set of *N* orthonormal states {|*n*〉_*A*_} forming the basis of the Hilbert space ${\mathscr{H}}_{A}$ which can be separated into two subsets ${\mathscr{H}}_{A}=\mathcal {W}\cup {\mathscr{L}}$, the set of rewarded states $\mathcal {W}$ and the set of losing states ${\mathscr{L}}$ with $\mathcal {W}\cap {\mathscr{L}}=\emptyset $. Information about rewarded states can be obtained by querying phase-flip oracles

48 $$ O_k=P_{\mathcal{W}_k}-P_{\mathcal{L}_k}. $$

where $P_{\mathcal {W}_{k}}$ and $P_{{\mathscr{L}}_{k}}$ denote projectors on some subspaces $\mathcal {W}_{k}$ and ${\mathscr{L}}_{k}$ forming together again the complete Hilbert space ${\mathscr{H}}_{A}=\mathcal {W}_{k}\cup {\mathscr{L}}_{k}$ with $\mathcal {W}_{k}\cap {\mathscr{L}}_{k}=\emptyset $. For standard search problems we have $\mathcal {W}_{k}=\mathcal {W} \forall k$ and ${\mathscr{L}}_{k}={\mathscr{L}} \forall k$. However, for more general search problems such as the here considered changing oracle problem, the subspaces $\mathcal {W}_{k}$ and ${\mathscr{L}}_{k}$ might differ from query to query.

Our goal is to find any state ${|n\rangle }_{A}\in \mathcal {W}$ with the help of maximally *K* oracle queries. All possible search strategies can be represented via unitary operations and pure initial states since it is possible to purify any search strategy by going to a larger Hilbert space ${\mathscr{H}}_{AB}={\mathscr{H}}_{A}\otimes {\mathscr{H}}_{B}$ and defining the generalize operators

49

To avoid a notation with over boarding indices, we skip the labels indicating the different subspace the operators/unitaries are working on if they are not crucial. Operators with a subspace index, such as e.g. *σ*_*A*_ acting on a state from a larger Hilbert space, e.g. |*ψ*〉_*A**B*_ are meant as short forms of the generalized operators defined similar to Eq. 49.

Any search strategy *T* to find a state ${|n\rangle }\in \mathcal {W}$ can be described via *T* = ({*U*_*k*_},|*ψ*(0)〉_*A**B*_) with a pure initial state |*ψ*(0)〉_*A**B*_ and unitaries {*U*_*k*_} acting on the combined Hilbert space ${\mathscr{H}}_{AB}$ leading after *K* oracle queries to the final state

50 $$ {|\psi(K)\rangle}_{AB}=U_KO_{K}{\cdots} U_2O_2U_{1} O_{1} {|\psi(0)\rangle}_{AB} $$

and a consecutive projective measurement. Without loss of generality, we apply first an oracle query since any unitary *U*_0_ applied before can be subsumed into the initial state. The probability *p*_*T*_ to identify a rewarded state correctly for a given strategy *T* and set of oracles {*O*_*k*_} is then given by

51 $$ p_T=\text{Tr }\left( P_{\mathcal{W}}{|\psi(K)\rangle}{\langle\psi(K)|}\right). $$

Let *σ* denote a permutation operator acting on ${\mathscr{H}}_{A}$ and Σ_*A*_ denoting the group of operators of all possible permutations. The average reward probability $\bar {p}_{T}$ of the strategy *T* is defined via

52 $$ \bar{p}_T=\frac{1}{N!}\sum\limits_{\sigma \in {\Sigma}_{A}}p_T(\sigma) =\frac{1}{N!}\sum\limits_{\sigma \in {\Sigma}_{A}}\text{Tr }\left[\sigma P_{\mathcal{W}}\sigma^{\dagger} {|\psi(K,\sigma)\rangle}{\langle\psi(K,\sigma)|}\right] $$

with

53 $$ {|\psi(K,\sigma)\rangle}_{AB}=U_{K}\sigma O_{K}\sigma^{\dagger}{\cdots} U_{1} \sigma O_{1}\sigma^{\dagger} {|\psi(0)\rangle}_{AB} $$

being the resulting state if we substitute every oracle *O*_*k*_ by *σ**O*_*k*_*σ*^‡^.

For every search strategy *T*_*A**B*_ = ({*U*_*k*, *A**B*_},|*ψ*(0)〉_*A**B*_), we can define an averaged strategy $\bar {T}_{ABC}$ via

### **Definition 1**

The averaged strategy $\bar {T}_{ABC}=(\lbrace {\bar {U}_{k,ABC}}\rbrace ,{|\bar {\psi }(0)\rangle }_{ABC})$ of the strategy *T*_*A**B*_ = ({*U*_*k*, *A**B*_},|*ψ*(0)〉_*A**B*_) is defined via the averaged initial state

54 $$ {|\bar{\psi}(0)\rangle}_{ABC}=\frac{1}{\sqrt{N!}}\sum\limits_{\sigma_{\gamma} \in {\Sigma}_{A}} \sigma_{\gamma}^{\dagger}{|\psi(0)\rangle}_{AB}{|\gamma\rangle}_C. $$

and average unitaries

55 $$ \bar{U}_{k,ABC}=\sum\limits_{\sigma_{\gamma} \in {\Sigma}_{A}} \sigma_{\gamma}^{\dagger} U_{k,AB} \sigma_{\gamma} \otimes {|\gamma\rangle}_C{\langle\gamma|}. $$

Here, the states {|*γ*〉_*C*_} are given by an arbitrary orthonormal basis of a Hilbert space ${\mathscr{H}}_{C}$ with dimension *d*_*C*_ = *N*! acting as labels for the applied permutation operator *σ*_*γ*_ acting on ${\mathscr{H}}_{A}$.

The averaged strategy $\bar {T}$ has the following properties:

### **Lemma 3**

The success probability $p_{\bar {T}}(\sigma )$ of the averaged strategy $\bar {T}$ is equal to the average success probability $\bar {p}_{T}$ of the strategy *T* for every permutation *σ* ∈Σ_*A*_.

### *Proof*

The success probability $p_{\bar {T}}(\sigma )$ is given by

56 $$ p_{\bar{T}}(\sigma)=\text{Tr }_{ABC}\left[\sigma P_{\mathcal{W},ABC}\sigma^{\dagger} {|\bar{\psi}(K,\sigma)\rangle}{\langle\bar{\psi}(K,\sigma)|}\right]. $$

The state $\sigma ^{\dagger }{|\bar {\psi }(K,\sigma )\rangle }_{ABC}$ for *σ* ∈Σ_*A*_ is given by

57 $$ \begin{array}{@{}rcl@{}} \sigma^{\dagger}{|\bar{\psi}(K,\sigma)\rangle}_{ABC}&=&\frac{1}{\sqrt{N!}}\sum\limits_{\sigma_{\gamma}\in {\Sigma}_{A}}{\sigma}^{\dagger}{\sigma}_{\gamma}^{\dagger} U_{K}{\sigma}_{\gamma}\sigma O_{K}{\sigma}^{\dagger}{\cdots} {\sigma}_{\gamma}^{\dagger} U_{1} {\sigma}_{\gamma} \sigma O_{1}{\sigma}^{\dagger} {\sigma}_{\gamma}^{\dagger}{|\psi(0)\rangle}_{AB}{|\gamma\rangle}_{C} \end{array} $$

58 $$ \begin{array}{@{}rcl@{}} &=&\frac{1}{\sqrt{N!}}\sum\limits_{\tilde{\sigma}_{\gamma}\in {\Sigma}_{A}}\tilde{\sigma}_{\gamma}^{\dagger} U_{K}{\tilde{\sigma}_{\gamma}}O_{K}{\cdots} {\tilde{\sigma}_{\gamma}}^{\dagger} U_{1} \tilde{\sigma}_{\gamma} O_{1} {\tilde{\sigma}_{\gamma}}^{\dagger}{|\psi(0)\rangle}_{AB}{|\gamma\rangle}_{C} \end{array} $$

where we used

59 $$ \sigma\sum\limits_{\sigma_{\gamma} \in{\Sigma}_{A}}\sigma_{\gamma} =\sum\limits_{\tilde{\sigma}_{\gamma} \in{\Sigma}_{A}}\tilde{\sigma}_{\gamma} \quad \forall \sigma\in{\Sigma}_{A} $$

because Σ_*A*_ is a symmetric group. As a consequence, the application of the permutation *σ*^‡^ on ${|\bar {\psi }(K)\rangle }$ is equivalent to a relabeling of the permutations *σ*_*γ*_ such that we now apply the permutation $\tilde {\sigma }_{\gamma }^{\dagger }=\sigma ^{\dagger }\sigma _{\gamma }^{\dagger } $ instead of $\sigma _{\gamma }^{\dagger }$ if subsystem *C* is in state |*γ*〉_*C*_. However, these labels have been arbitrary and therefore we find for the success probability

60 $$ \begin{array}{@{}rcl@{}} p_{\bar{T}}(\sigma)&=&\text{Tr }_{ABC}\left[ P_{\mathcal{W},ABC}\sigma^{\dagger} {|\bar{\psi}(K,\sigma)\rangle}{\langle\bar{\psi}(K,\sigma)|}\sigma\right] \end{array} $$

61 $$ \begin{array}{@{}rcl@{}} &=&\frac{1}{N!}\sum\limits_{\tilde{\sigma} \in {\Sigma}_{\mathcal{H}_{A}}}\text{Tr }_{AB}\left[\tilde{\sigma}P_{\mathcal{W},AB}{\tilde{\sigma}}^{\dagger} {|\psi(K),\tilde{\sigma}\rangle}_{AB}{\langle\psi(K),\tilde{\sigma}|}_{AB} \right] \end{array} $$

62 $$ \begin{array}{@{}rcl@{}} &=&\bar{p}_{T} \end{array} $$

□

The relabeling can be formalized in the following way. We define the index $\tilde {\gamma }$ via $\sigma \sigma _{\gamma }= \sigma _{\tilde {\gamma }}$. Then, we can define the permutation *π*(*σ*) acting on ${\mathscr{H}}_{C}$ via

63 $$ \pi(\sigma){|\gamma\rangle}_C={|\tilde{\gamma}\rangle}_C $$

which then leads to the following lemma:

### **Lemma 4**

The averaged strategy $\bar {T}$ is permutation invariant under joined permutations *σ* ⊗ *π*(*σ*) ∀*σ* ∈Σ_*A*_ such that

64 $$ \begin{array}{@{}rcl@{}} [\bar{U}_{k},\sigma\otimes \pi(\sigma)]&=&0 \end{array} $$

65 $$ \begin{array}{@{}rcl@{}} \sigma\otimes \pi(\sigma){|\bar{\psi}(0)\rangle}&=&{|\bar{\psi}(0)\rangle}. \end{array} $$

### *Proof*

For the symmetric initial state ${|\bar {\psi }(0)\rangle }$, we find

66 $$ \begin{array}{@{}rcl@{}} \sigma\otimes\pi(\sigma){|\bar{\psi}(0)\rangle}_{ABC} &=& \sigma\otimes \pi(\sigma) \frac{1}{\sqrt{N!}}\sum\limits_{\sigma_{\gamma}\in{\Sigma}_{A}} \sigma_{\gamma}{|{\psi(0)}\rangle}_{AB}{|\gamma\rangle}_{C} \end{array} $$

67 $$ \begin{array}{@{}rcl@{}} &=& \frac{1}{\sqrt{N!}}\sum\limits_{\sigma_{\gamma} \in {\Sigma}_{A}} \sigma\sigma_{\gamma}{|\psi(0)\rangle}_{AB}\pi(\sigma){|\gamma\rangle}_{C} \end{array} $$

68 $$ \begin{array}{@{}rcl@{}} &=&\frac{1}{\sqrt{N!}}\sum\limits_{\sigma_{\tilde{\gamma}} \in {\Sigma}_{A}} \sigma_{\tilde{\gamma}}{|\psi(0)\rangle}_{AB}{|\tilde{\gamma}\rangle}_{C}={|\bar{\psi}(0)\rangle}_{ABC}. \end{array} $$

For the symmetric unitaries $\bar {U}_{k}$, we find

69 $$ \begin{array}{@{}rcl@{}} \sigma\otimes \pi(\sigma)\bar{U}_{k}{\sigma}^{\dagger}\otimes \pi^{\dagger}(\sigma)&=& \sigma\otimes \pi(\sigma)\left( \sum\limits_{\sigma_{\gamma} \in {\Sigma}_{A}} \sigma_{\gamma} U_{k,AB} \sigma_{\gamma}^{\dagger} \otimes {|\gamma\rangle}_{C}{\langle\gamma|}\right){\sigma}^{\dagger}\otimes \pi^{\dagger}(\sigma) \end{array} $$

70 $$ \begin{array}{@{}rcl@{}} &=&\sum\limits_{\sigma_{\tilde{\gamma}} \in {\Sigma}_{A}} \sigma_{\tilde{\gamma}} U_{k,AB} \sigma_{\tilde{\gamma}}^{\dagger} \otimes {|\tilde{\gamma}\rangle}_{C}{\langle\tilde{\gamma}|} \end{array} $$

71 $$ \begin{array}{@{}rcl@{}} &=&\bar{U}_{k}. \end{array} $$

$[\bar {U}_{k},\sigma \otimes \pi (\sigma )]=0$ follows immediately since permutation operators are unitary. □

As a consequence of Lemmas 3 and 4, we can limit the search for the best strategy *T*, optimizing $\bar {p}_{T}$, to averaged strategies $\bar {T}$ which also optimize the worst case probability $\underset {\sigma }{\text {min }}p_{T}(\sigma )$ and leads to certain symmetries as outlined in Appendix C.

## Appendix C: Symmetry investigations for the changing oracle problem

In the following, we consider a search problem, where the oracle *O*_*k*_ changes at a certain time step. Thus, we can separate the search into two phases. The first phase contains *K* oracle queries to oracle $\tilde {O}=O_{k}$ for 1 ≤ *k* ≤ *K* with rewarded space $\tilde {\mathcal {W}}$ and losing space $\tilde {{\mathscr{L}}}$. Then, the oracle changes to *O* = *O*_*k*_ for *K* < *k* ≤ *J* with the new rewarded space ${\mathcal {W}}$ and losing space ${{\mathscr{L}}}$ and the search is continued by another *J* queries to *O*. In addition, we restrict the problem to monotonically increasing rewarded spaces that is the rewarded space $\tilde {\mathcal {W}}$ of the first phase is a subset $\tilde {\mathcal {W}}\subseteq \mathcal {W}$ of the rewarded space $\mathcal {W}$ of the second oracle *O*. This automatically leads to $\tilde {{\mathscr{L}}}\supseteq {\mathscr{L}}$.

In the following, we investigate the appearing symmetries occurring during the first and second phase when applying averaged search strategies $\bar {T}$ to this problem. Since we only consider averaged strategies and thus averaged unitaries $\bar {U}_{k}$ and states ${|\bar {\psi }(k)\rangle }$, we omit the bar on all states and unitaries in this section to simplify the notation.

In the following, we investigate the symmetry properties of the states

72 $$ \begin{array}{@{}rcl@{}} {|\psi(K)\rangle}&=&U_{K} O_{K} {\cdots} U_{1} O_{1}{|\psi(0)\rangle} \end{array} $$

73 $$ \begin{array}{@{}rcl@{}} {|\psi(K+J)\rangle}&=&U_{K+J}O_{K+J}{\cdots} U_{K+1}O_{K+1}{|\psi(K)\rangle} \end{array} $$

at the end of the first and the second phase. This will allow us to determine an upper bound for the average success probability *p*.

We define the set of permutations (operators) ${\Sigma }_{\tilde {O}}={\Sigma }_{\tilde {\mathcal {W}}}\cup {\Sigma }_{\tilde {{\mathscr{L}}}}$ as the complete set of permutations operators which leave the rewarded space $\tilde {\mathcal {W}}$ and losing space $\tilde {{\mathscr{L}}}$ invariant. As a consequence, we find $[\tilde {O},\sigma ]=0 \forall \sigma \in {\Sigma }_{\tilde {O}}$. The initial state |*ψ*(0)〉 and all unitaries *U*_*k*_ and $\tilde {O}_{k}$ during the first phase are permutation invariant under *σ* ⊗ *π*(*σ*) $ \forall \sigma \in {\Sigma }_{\tilde {O}}$ since ${\Sigma }_{\tilde {O}}\subseteq {\Sigma }_{{\mathscr{H}}_{A}}$. Thus, the state |*ψ*(*K*)〉 at the end of the first phase is also permutation invariant under *σ* ⊗ *π*(*σ*) $ \forall \sigma \in {\Sigma }_{\tilde {O}}$.

To determine the symmetry properties of |*ψ*(*K* + *J*)〉, we need to investigate how the rewarded and not-rewarded components of |*ψ*(*K*)〉 changes when we change the oracle. We define the normalized rewarded ${|\tilde {w}\rangle }$ and not-rewarded component ${|\tilde {\ell }\rangle }$ of |*ψ*(*K*)〉 via

74 $$ \begin{array}{@{}rcl@{}} \cos\alpha{|\tilde{\ell}\rangle}&=&P_{\tilde{\mathcal{L}}}{|\psi(K)\rangle} \end{array} $$

75 $$ \begin{array}{@{}rcl@{}} \sin\alpha{|\tilde{w}\rangle}&=&P_{\tilde{\mathcal{W}}}{|\psi(K)\rangle} \end{array} $$

with ${\cos \limits } \alpha = |P_{\tilde {{\mathscr{L}}}}{|\psi (K)\rangle }|$. As a consequence, |*ψ*(*K*)〉 can be decomposed via

76 $$ {|\psi(K)\rangle}=\cos \alpha {|\tilde{\ell}\rangle}+\sin{\alpha}{|\tilde{w}\rangle}.  $$

The components ${|\tilde {w}\rangle }$ and ${|\tilde {\ell }\rangle }$ are permutation invariant under *σ* ⊗ *π*(*σ*) $ \forall \sigma \in {\Sigma }_{\tilde {O}}$ because the projectors $P_{\tilde {\mathcal {W}}}$ and $P_{\tilde {{\mathscr{L}}}}$ as well as |*ψ*(*K*)〉 are permutation invariant.

Let us now investigate the rewarded and not-rewarded components at the beginning of the second phase. The initial state of the second phase is given by |*ψ*(*K*)〉. Its component ${|\tilde {w}\rangle }$ is also a rewarded component according to the second oracle *O* such that $P_{\mathcal {W}}{|\tilde {w}\rangle }={|\tilde {w}\rangle }$. However, ${|\tilde {\ell }\rangle }$ contains both rewarded and not-rewarded components

77 $$ \begin{array}{@{}rcl@{}} \cos \beta{|\ell\rangle}&=&P_{{\mathcal{L}}}{|\tilde{\ell}\rangle} \end{array} $$

78 $$ \begin{array}{@{}rcl@{}} \sin \beta{|w_{+}\rangle}&=&P_{{\mathcal{W}}}{|\tilde{\ell}\rangle} \end{array} $$

with ${\cos \limits } \beta = |P_{{{\mathscr{L}}}}{|\tilde {\ell }\rangle }|$. Note, that ${|w_{+}\rangle } \in \mathcal {W}_{+}=\tilde {{\mathscr{L}}}\cap \mathcal {W}$ and thus ${|w_{+}\rangle }\perp {|\tilde {w}\rangle }$. Therefore, we can divide the state |*ψ*(*K*)〉 into three orthogonal components via

79 $$ {|\psi(K)\rangle}=\sin\alpha {|\tilde{w}\rangle}+\cos\alpha \left( \sin \beta {|w_+\rangle}+\cos \beta {|\ell\rangle}\right).  $$

The angle *β* is given by

80 $$ \sin \beta = \sqrt{\frac{n_+}{n_++n_{\ell}}}  $$

where *n*_+_ denotes the dimension of $\mathcal {W}_{+}$ and *n*_*ℓ*_ the dimension of ${\mathscr{L}}$ (see Appendix D).

Let us now invest the symmetries of |*ψ*(*K*)〉 with respect to permutations *σ* ⊗ *π*(*σ*) ∀*σ* ∈Σ_*O*_ which leave the second oracle *O* invariant. Let $P_{\mathcal {S}}$ be the projector onto the symmetric subspace which can be written as

81 $$ P_{\mathcal{S}}= \sum\limits_{{|s\rangle}}{|s\rangle}{\langle s|} \quad \text{with} \quad \sigma\otimes \pi(\sigma){|s\rangle}={|s\rangle}\quad \forall \sigma\in {\Sigma}_{O} $$

where {|*s*〉} forms an orthonormal basis of the symmetric subspace. Then, we can define the symmetric component

82 $$ \cos \varepsilon{|\phi_{s}\rangle}=P_{\mathcal{S}}{|\psi(K)\rangle}  $$

and its complement

83

with ${\cos \limits } \varepsilon =|P_{\mathcal {S}}{|\psi (K)\rangle }|$. The state |*ℓ*〉 is permutation invariant under *σ* ⊗ *π*(*σ*) ∀*σ*_*A*_ ∈Σ_*O*_ since ${\mathscr{L}}\subseteq \tilde {{\mathscr{L}}}$ such that $P_{\mathcal {S}}{|\ell \rangle }={|\ell \rangle }$. However, the (not normalized) rewarded component $\sin \limits \alpha {|\tilde {w}\rangle }+\cos \limits \alpha {\sin \limits } \beta {|w_{+}\rangle }$ is not necessarily permutation invariant under *σ* ⊗ *π*(*σ*) ∀*σ* ∈Σ_*O*_. As a consequence, there might exist a non-vanishing component |*ϕ*_⊥_〉; however, this component lies within the rewarded space $\mathcal {W}$ such that

84

The symmetric component |*ϕ*_*S*_〉 can be decomposed into a rewarded and a not-rewarded component

85 $$ \begin{array}{@{}rcl@{}} \cos \varepsilon \sin \phi{|w_{s}\rangle}&=&P_{\mathcal{W}}P_{\mathcal{S}}{|\psi(K)\rangle} \end{array} $$

86 $$ \begin{array}{@{}rcl@{}} \cos \varepsilon \cos \phi{|\ell_{s}\rangle}&=&P_{\mathcal{L}}P_{\mathcal{S}}{|\psi(K)\rangle}=\cos \varepsilon \cos \phi{|\ell\rangle} \end{array} $$

with $\cos \limits \varepsilon {\sin \limits } \phi =|P_{\mathcal {W}}P_{\mathcal {S}}{|\psi (K)\rangle }|$. Thus, the state |*ψ*(*K*)〉 can be separated into the following three orthogonal components

87 $$ {|\psi(K)\rangle}=\cos{\varepsilon}\left( \sin{\phi}{|w_{s}\rangle}+\cos \phi{|\ell\rangle}\right)+\sin{\varepsilon}{|w_{\perp} \rangle}. $$

A comparison with Eq. 79 leads to the following identities

88 $$ \begin{array}{@{}rcl@{}} \cos \varepsilon \cos\phi &=& {\langle \ell|\psi(K)\rangle}= \cos \alpha \cos \beta \end{array} $$

89 $$ \begin{array}{@{}rcl@{}} \cos \varepsilon \sin \phi&=&{\langle w_{s}|\psi(K)\rangle}= \sin\alpha {\langle w_{s}|\tilde{w}\rangle}+\cos\alpha\sin\beta{\langle w_{s}|{w}_{+}\rangle} \end{array} $$

90 $$ \begin{array}{@{}rcl@{}} \sin\varepsilon &=&{\langle w_{\perp}|\psi(K)\rangle}= \sin\alpha {\langle w_{\perp}|\tilde{w}\rangle}+\cos\alpha\sin\beta{\langle w_{\perp}|{w}_{+}\rangle}. \end{array} $$

Note, all appearing scalar products are real due to the definition of |*w*_*s*_〉 and |*w*_⊥_〉 and they are upper bounded via

91 $$ \begin{array}{@{}rcl@{}} |{\langle w_{s}|\tilde{w}\rangle}|&\leq& \sqrt{\frac{\tilde{n}}{\tilde{n}+n_{+}}} \end{array} $$

92 $$ \begin{array}{@{}rcl@{}} |{\langle w_{s}|{w}_{+}\rangle}|&\leq& \sqrt{\frac{{n}_{+}}{{n}_{\ell}+n_{+}}}. \end{array} $$

As a consequence, the angle *ϕ* is upper bounded by the angle *α* via

93 $$ \tan \phi \leq \tan \alpha \sqrt{\frac{\tilde{n}(n_++n_{\ell})}{(\tilde{n}+n_+)n_{\ell}}}+\sqrt{\frac{n_+^{2}}{(\tilde{n}+n_+)n_{\ell}}}.  $$

Let us investigate the time evolution during the second phase. We denote with

94 $$ V=U_{K+J}O {\cdots} U_{K+1}O $$

a unitary which described the complete time evolution during the second phase. The unitary *V* commutes with the projector $P_{\mathcal {S}}$ as the following considerations will prove. There exist a joined eigenbasis of *V* and *σ* ⊗ *π*(*σ*) ∀*σ* ∈Σ_*O*_ since [*V*, *σ* ⊗ *π*(*σ*)] = 0. Let {|*v*_*x*_〉} be an eigenbasis of *V* and wlog we assume that the first *f* states of this basis form the symmetric subspace such that

95 $$ \sigma\otimes \pi(\sigma){|v_x\rangle}={|v_x\rangle} \quad \forall \sigma \in {\Sigma}_O \text{ and }1\leq x\leq f. $$

As a consequence, we find

96 $$ P_{\mathcal{S}}V=\sum\limits_{x=1}^f {|v_x\rangle}{\langle v_x|}\sum\limits_{y} \lambda_{y} {|v_{y}\rangle}{\langle v_{y}|}=\sum\limits_{x=1}^f \lambda_x{|v_x\rangle}{\langle v_x|}=VP_{\mathcal{S}} $$

where *λ*_*y*_ denote the eigenvalues of *V*. Thus, the time evolution of the symmetric component *V* |*ϕ*_*S*_〉 stays a symmetric state with

97 $$ P_{\mathcal{S}}V{|\phi_S\rangle}=VP_{\mathcal{S}}{|\phi_S\rangle}=V{|\phi_S\rangle} $$

whereas *V* |*ϕ*_⊥_〉 stays orthogonal to this subspace since

98 $$ P_{\mathcal{S}}V{|\phi_{\perp} \rangle}=VP_{\mathcal{S}}{|\phi_{\perp} \rangle}=0 $$

and thus the symmetric part and the orthogonal part do not mix.

The reward probability of |*ψ*(*K* + *J*)〉 can be decomposed into a symmetric part and a part orthogonal to it via

99

100 $$ \begin{array}{@{}rcl@{}} \quad\quad\quad\quad\quad\quad\quad\quad\quad\quad\quad\quad\quad{\kern4pt} &=&\cos^{2} \varepsilon \text{Tr }\left[P_{\mathcal{W}} V{|\phi_{s}\rangle}{\langle\phi_{s}|}V^{\dagger}\right] \\ \quad\quad\quad\quad\quad\quad\quad\quad\quad\quad\quad\quad\quad &&+\sin^{2} \varepsilon \text{Tr }\left[P_{\mathcal{W}} V{|\phi_{\perp}\rangle}{\langle\phi_{\perp}|}V^{\dagger}\right] \end{array} $$

where we used $[P_{\mathcal {W}},P_{\mathcal {S}}]=0$ which follows directly from $[P_{\mathcal {W}},\sigma \otimes \pi (\sigma )]=0$ ∀*σ* ∈Σ_*O*_ and $P_{S}={P_{S}^{2}}$.

## Appendix D: Determining the angle *β*

In the following, we give a more detailed derivation of Eq. 80 for determining *β* defined via

101 $$ \sin^{2} \beta = {\langle\tilde{\ell}|}P_{\mathcal{W}}{|\tilde{\ell}\rangle}. $$

Let wlog {|*j*〉_*A*_} with 1 ≤ *j* ≤ *n*_+_ + *n*_*ℓ*_ be a basis of the losing space $\tilde {{\mathscr{L}}}_{A}$. The state ${|\tilde {\ell }\rangle }_{ABC}$ can then be written as

102 $$ {|\tilde{\ell}\rangle}=\sum\limits_{j=1}^{n_++n_{\ell}}\xi_j {|j\rangle}_{A}{|\gamma_j\rangle}_{BC} $$

with some arbitrary normalized states |*γ*_*j*_〉_*B**C*_. The probability for each state |*j*〉_*A*_ is given by

103

However, the state ${|\tilde {\ell }\rangle }$ is permutation invariant $\forall \sigma \in {\Sigma }_{\tilde {{\mathscr{L}}}}$ such that

104 $$ \begin{array}{@{}rcl@{}} \sigma\otimes\pi(\sigma){|\tilde{\ell}\rangle}&=&\sum\limits_{j=1}^{n_{+}+n_{\ell}}\xi_{j} {|j^{\prime}(\sigma)\rangle}_{A}{|\gamma^{\prime}_{j}\rangle}_{BC}={|\tilde{\ell}\rangle} \end{array} $$

with . As a consequence, we find for the probabilities

105

and due to normalization *p*_*j*_ = 1/(*n*_+_ + *n*_*ℓ*_). Since there exist *n*_+_ orthonormal states within the subspace $\mathcal {W}_{+}=\mathcal {W}\cap \tilde {{\mathscr{L}}}$ we find

106 $$ \sin^{2}\beta = \frac{n_+}{n_++n_{\ell}}. $$

## Appendix E: Optimality proof of Grover’s algorithm for multiple rewarded items

The optimality proof of Grover’s algorithm for oracles with a single rewarded item by Zalka (1999) consist of two parts given by the inequality

107 $$ 2N-2\sqrt{Np}-2\sqrt{N(N-1)(1-p)}\leq \sum\limits_{y=1}^N|| {|\phi_{J}\rangle}-{|\phi_{J}^y\rangle}||^{2}\leq 4N\sin^{2}(J\psi). $$

Here, *N* is the number of items, *p* the success probability to identify the single rewarded item *y* correctly, *J* the maximal number of oracle queries and the angle *ψ* is defined via $\sin \limits ^{2}\psi =1/N$. The two quantum states |*ϕ*_*j*_〉 and ${|{\phi _{j}^{y}}\rangle }$ are defined via

108 $$ \begin{array}{@{}rcl@{}} {|\phi_{j}\rangle}=V^{j}{|\phi\rangle} \end{array} $$

109 $$ \begin{array}{@{}rcl@{}} {|{\phi_{j}^{y}}\rangle}={V^{j}_{y}}{|\phi\rangle} \end{array} $$

where |*ϕ*〉 is some arbitrary state, ${V_{y}^{j}}$ a unitary of the form Eq. 94 based on *j* queries to the oracle *O*_*y*_ and *V*^*j*^ is a unitary based on *j* queries to an empty oracle. The optimality of Grover’s algorithm follows from the proof of both inequalities and the fact that Grover’s algorithm saturates both.

We generalize the results from Zalka by going to oracles *O*_*y*_ which mark exactly *n* items out of *N* items as rewarded. In this case, *y* is now a label for the rewarded space $\mathcal {W}_{y}$ and there exist now $D={N \choose n}$ different oracles. The success probability *p* now denotes the probability to identify any rewarded item ${|z\rangle }\in \mathcal {W}_{y}$ correctly. For a random guess, this probability is given by $\sin \limits ^{2}\nu =n/N$. As a consequence, Eq. 107 can be generalized to

110 $$ 2D-2D\sqrt{p\frac{n}{N}}-2D\sqrt{(1-p)\left( 1-\frac{n}{N}\right)}\leq \sum\limits_{y=1}^D|| {|\phi_{J}\rangle}-{|\phi_{J}^y\rangle}||^{2}\leq 4D\sin^{2}(J\nu)  $$

which we will proof in the following and is equal to Eq. 107 for *n* = 1. Again, Grover’s algorithm saturates these bounds.

We start with the right inequality and proof the following lemma

### **Lemma 5**

The maximal difference between |*ϕ*_*J*_〉 and ${|{\phi _{j}^{y}}\rangle }$ achievable with *J* oracle queries averaged over all possible oracles with *n* rewarded items is given by

111 $$ \frac{1}{D}\sum\limits_{y=1}^D|| {|\phi_{J}\rangle}-{|\phi_{J}^y\rangle}||^{2}\leq 4\sin^{2}(J\psi)=2[1-\cos(2J\nu)]  $$

with $\sin \limits ^{2}\nu =n/N$.

### *Proof*

This Lemma follows directly from the optimality proof of Grover’s algorithm given in Zalka (1999) by generalizing the sum overall possible oracles which mark only one item *y* to all possible oracles which mark *n* items. In the following, we do not reproduce every step from Ref. (Zalka 1999) but concentrate only on steps where the generalization from one rewarded item to several rewarded items makes a difference. Following Ref. Zalka (1999) we find (Eq. 22)

112 $$ \frac{1}{D}\sum\limits_{y=1}^D || {|\phi_{J}\rangle}-{|\phi_{J}^y\rangle}||^{2} \leq Df(x)  $$

with the argument

113 $$ x=\frac{4J}{D}\sum\limits_{y=1}^D\sum\limits_{j=1}^J ||P_{\mathcal{W}_{y}}{|\phi_j\rangle}||^{2} $$

and $P_{\mathcal {W}_{y}}$ the projector onto the rewarded space of oracle *y*.The function *f*(*x*) is defined in Zalka (1999) via

114 $$ f\left( x=4J^{2}\sin^{2}\nu\right)= 4\sin^{2}(J\nu). $$

Every state ${|z\rangle }\in {\mathscr{H}}_{A}$ is part of the rewarded space $\mathcal {W}_{y}$ for exactly $d={{N-1}\choose {n-1}}$ different oracles. As a consequence, the argument *x* of the function *f* in Eq. 112 is given by

115 $$ \begin{array}{@{}rcl@{}} x=\frac{4J}{D}\sum\limits_{y=1}^{D}\sum\limits_{j=1}^{J} ||P_{\mathcal{W}_{y}}{|\phi_{j}\rangle}||^{2} &=&\frac{4J}{D}\sum\limits_{j=1}^{J}\sum\limits_{y=1}^{D} \sum\limits_{z\in \mathcal{W}_{y}}||{\langle z|\phi_{j}\rangle}||^{2} \end{array} $$

116 $$ \begin{array}{@{}rcl@{}} & &= 4J\sum\limits_{j=1}^{J} \frac{d}{D} \sum\limits_{z=1}^{N} ||{\langle z|\phi_{j}\rangle}||^{2}. \end{array} $$

The sum over all states |*z*〉 sums up to unity leading to

117 $$ \frac{4J}{D}\sum\limits_{y=1}^D\sum\limits_{j=1}^J ||P_{\mathcal{W}_{y}}{|\phi_j\rangle}||^{2} = 4J^{2}\frac{n}{N}=4J^{2}\sin^{2} \nu $$

where we used *d*/*D* = *n*/*N*. □

Grover’s algorithm saturates this inequality since we find for this algorithm

118 $$ \begin{array}{@{}rcl@{}} {|\phi\rangle}&=&\frac{1}{\sqrt{N}}\sum\limits_{z=1}^{N} {|z\rangle}=\cos \nu {|\ell\rangle}+ \sin \nu{|w\rangle} \end{array} $$

119 $$ \begin{array}{@{}rcl@{}} {|{\phi_{J}^{y}}\rangle}&=&\cos [(2J+1)\nu] {|\ell\rangle}+ \sin [(2J+1)\nu] {|w\rangle} \end{array} $$

120 $$ \begin{array}{@{}rcl@{}} {|\phi_{J}\rangle}&=&\cos \nu {|\ell\rangle}+ \sin \nu {|w\rangle} \end{array} $$

leading to

121 $$ \begin{array}{@{}rcl@{}} \frac{1}{D}\sum\limits_{y=1}^{D}|| {|\phi_{J}\rangle}-{|{\phi_{J}^{y}}\rangle}||^{2}&=&2-2 \sin[(2J+1)\nu]\sin(\nu)-2\cos[(2J+1)\nu]\cos(\nu) \end{array} $$

122 $$ \begin{array}{@{}rcl@{}} &=&2[ 1-\cos(2J\nu)]. \end{array} $$

The right side of Eq. 110 is govern by the lemma

### **Lemma 6**

The average success probability *p* to identify any item $z\in \mathcal {W}_{y}$ out of the rewarded space $\mathcal {W}_{y}$ of the oracle *O*_*y*_ with 1 ≤ *y* ≤ *D* given the states ${\phi ^{y}_{J}}$ average over all oracles is upper bounded by

123 $$ 2-2\sqrt{p\frac{n}{N}}-2\sqrt{(1-p)\left( 1-\frac{n}{N}\right)}\leq \frac{1}{D}\sum\limits_{y=1}^D|| {|\phi_{J}\rangle}-{|\phi^y_{J}\rangle}||^{2} $$

### *Proof*

Again, in order to proof this lemma, we follow the proof in Zalka (1999) and only point out the generalizations we have to make when going form *n* = 1 rewarded state to *n* > 1 rewarded states. Similar to (Zalka 1999), we write the states

124 $$ {|\phi^y_{J}\rangle}=\sum\limits_{x=1}^X c_x^y{|x\rangle} \quad {|\phi_{J}\rangle}=\sum\limits_{x=1}^X c_x{|x\rangle} $$

via some orthonormal basis {|*x*〉} of some Hilbert space with dimension *X*. The optimal procedure to identify a rewarded item |*z*〉 is to perform projective measurements (see Ref. Zalka (1999)). Let {|*x*〉} be the measurement basis and we denote with *X*_*z*_ the subspace containing all states |*x*〉 which correctly denote that |*z*〉 is a rewarded item. As a consequence, the success probability *p*_*y*_ if the unknown oracle is given by *O*_*y*_ is determined via

125 $$ p_{y}=\sum\limits_{z\in \mathcal{W}_{y}}\sum\limits_{x\in X_z}|c_x^y|^{2}. $$

Similar, we can define a success probability *a*_*y*_ for the state |*ϕ*_*J*_〉 via (compare Eq. A7 in Zalka (1999))

126 $$ a_{y}=\sum\limits_{z\in \mathcal{W}_{y}}\sum\limits_{x\in X_z}|c_x|^{2}. $$

In order to proof (6), Zalka determines the minimal distance an arbitrary state |*ϕ*_*y*_〉 with success probability *p*_*y*_ needs to have from a given state |*ζ*_*y*_〉 with success probability *a*_*y*_. This minimal distance is given (compare Eq. A8 in Zalka (1999)) by

127 $$ ||{|\phi_{y}\rangle}-{|\zeta_{y}\rangle}||^{2}\geq 2-2\left( \sqrt{p_ya_{y}}+\sqrt{(1-p_{y})(1-a_{y})}\right).  $$

The minimum of

128 $$ \frac{1}{D}\sum\limits_{y=1}^D|| {|\phi_{y}\rangle}-{|\zeta_{y}\rangle}||^{2} $$

for all possibly states |*ζ*_*y*_〉 and success probabilities *p*_*y*_ is reached if all *p*_*y*_ = *p* and *a*_*y*_ = *a* (see Zalka (1999)). Due to normalization we find

129 $$ \sum\limits_{y=1}^D a_{y}=d\sum\limits_{x=1}^X |c_x|=d $$

where we have used that each item |*z*〉 belongs to the rewarded space of $d={{N-1}\choose {n-1}}$ different oracles. As a consequence, the minimum is achieved for *a*_*y*_ = *d*/*D* = *n*/*N* (see discussion before Eq. A10 in Zalka (1999)) leading finally to the modification of Eq. A10 Zalka (1999) to

130 $$ \frac{1}{D}\sum\limits_{y=1}^D|| {|\phi_{J}\rangle}-{|\phi^y_{J}\rangle}||^{2} \geq 2-2\sqrt{p\frac{n}{N}}-2\sqrt{(1-p)\left( 1-\frac{n}{N}\right)} $$

which gives us directly Lemma 6. Also this bound is satisfied by Grover’s algorithm. □

The above-stated optimality proof of Grover’s algorithm can be easily generalized to situation where we start in a state |*ζ*_*y*_〉 with success probability $a_{y}=a=\sin \limits ^{2} \phi $ and try to optimize the success probability *p*_*y*_ of ${V_{y}^{J}}{|\zeta _{y}\rangle }$ with the help of maximal *J* oracle queries. Lemma 5 is independent from the initial state and can therefore directly be applied. From Eq. 127 we find

131 $$ \frac{1}{D}\sum\limits_{y=1}^D|| V_{y}^J{|\zeta_{y}\rangle}-{|\zeta_{y}\rangle}||^{2}\geq 2-2\sum\limits_{y}^D \sqrt{p_{y}\sin^{2}\phi}\sqrt{(1-p_{y})\cos^{2}\phi} $$

which is minimal if *p*_*y*_ = *p*∀*y*. Thus, we find

132 $$ \frac{1}{D}\sum\limits_{y=1}^D|| V_{y}^J{|\zeta_{y}\rangle}-{|\zeta_{y}\rangle}||^{2}\geq 2-2\sqrt{p\sin^{2}\phi}\sqrt{(1-p)\cos^{2}\phi}.  $$

Lemma 5 and Eq. 132 can be simultaneously saturated by starting in a state

133 $$ {|\zeta_{s}\rangle}=\sin \phi \frac{1}{\sqrt{|\mathcal{W}_{y}}|}\sum\limits_{{|z\rangle}\in \mathcal{W}_{y}}{|z\rangle} + \cos \phi \frac{1}{\sqrt{|\mathcal{L}_{y}|}}\sum\limits_{{|z\rangle}\in \mathcal{L}_{y}}{|z\rangle} $$

and performing Grover iterations via the unitary

134

135 $$ {|\phi\rangle}=\frac{1}{\sqrt{N}}\sum\limits_{z=1}^{N} {|z\rangle}. $$

Applying *V*^*J*^ with an empty oracle on |*ζ*_*s*_〉 does not change the success probability *a*_*y*_ leading to a maximal success probability $p=\sin \limits ^{2}(\phi +\nu )$ with $\sin \limits ^{2}\nu =n/N$.

## Appendix F: Minimal cost

In the following, we consider a data base search where the oracle $\tilde {O}$ is exchange at time *t*_0_ by a new oracle *O* with rewarded space $W\supseteq \tilde {W}$. Before *t*_0_, several Grover iterations $\tilde {G}_{\psi }$ have been applied and created the state |*ψ*(*K*)〉 as given in Eq. 32. Then, the oracle changes. The probability to project |*ψ*(*K*)〉 onto a target state directly after *t*_0_ is given by

136 $$ p_{K}= || P_{\mathcal{W}} {|\psi(K)\rangle}||^{2}=\sin^{2}\varepsilon+\cos^{2}\varepsilon\sin^{2} \phi_{K}. $$

In the following, we want to minimize the cost to find a target state. A typical cost for Grover’s algorithm with *j* steps is given by *C*(*j*) = 2*j* + 1 (see e.g. Dunjko et al. (2016) and Sriarunothai et al. (2019)).

The standard Grover algorithm considers only static oracles. Hence, the coherent evolution has to be terminated when the oracle changes. Thus in the standard case, we perform a measurement to finish the coherent time evolution based on $\tilde {G}_{\psi }$. If no target state has been found, we continue by performing Grover iterations with the new oracle described by *G*_*ψ*_. The average cost *C*_*s*_ for the standard Grover algorithm is then given by

137 $$ C_{s}=1+(1-p_{K})\frac{2j+1}{\sin^{2}[(2j+1)\nu]}, $$

for a fixed number of *j* steps and $\sin \limits ^{2}\nu =|\mathcal {W}|/N$. Here, we neglected all costs accumulated before *t*_0_. The minimum cost is achieved for $\tan [(2j+1)\nu ]\approx 2(2j+1)\nu $ which can be solved numerically. The numerical solution corresponds to apply Grover’s algorithm until a probability to find a target is approximately $\sin \limits ^{2} [(2j+1)\nu ]\approx 0.844$ and leads to the average cost of

138 $$ C_{s}^{\text{opti}}\approx 1+(1-p_{K})\frac{1.38}{\nu}.  $$

We generalize Grover’s algorithm by continuing the coherent time evolution after the oracle has changed. The cost for continuing the Grover search with *j* applications of *G*_*ψ*_ is given by (2*j* + 1). A consecutive measurement will project the resulting state |*ψ*(*K* + *j*)〉 onto a target state with probability (see Eq. 36)

139 $$ p_{K+j}=\sin^{2}\varepsilon_{K}+\cos^{2}\varepsilon_{K}\sin^{2}(2j\nu+\phi_k). $$

If we do not find a target, we continue with a standard Grover search based on the new oracle leading to the same expected average cost of 1.38/*ν*. Thus, we expect in total an average cost of

140 $$ C_{g}\approx (2j+1)+(1-p_{K+j})\frac{1.38}{\nu} $$

if we continue the coherent time evolution by another *j* Grover iterations *G*_*ψ*_ although the oracle has changed before we perform a measurement. By migrating *j* into a real valued variable, we can optimize *C*_*g*_ over *j*. Extremal points of *C*_*g*_ can be found for values of *j* satisfying

141 $$ \sin\left[4j\nu+2\phi_{K}\right]= \frac{1}{1.38 \cos^{2}\varepsilon_{K}}. $$

For $\cos \limits ^{2}\varepsilon _{K}>1/1.38$, we find a maximum of *C*_*g*_ at

142 $$ j\approx \frac{1}{4\nu}\left[\arcsin\left( \frac{1}{1.38 \cos^{2}\varepsilon_{K}}\right)-2\phi_{K}\right] $$

and a minimum at

143 $$ j\approx \frac{1}{4\nu}\left[\pi-\arcsin\left( \frac{1}{1.38 \cos^{2}\varepsilon_{K}}\right)-2\phi_{K}\right]. $$

This minimum corresponds to a probability to find a target state at the first measurement given by

144 $$ p_{\text{opti}}(\varepsilon_{K})= \frac{1}{2}\left( 1+\sin^{2}\varepsilon_{K}-\sqrt{\cos^4\varepsilon_{K}-\frac{1}{1.38^{2}}}\right) $$

which is independent of *ν*. As a consequence, the average cost of a continuous Grover algorithm optimized over j can be written as

145 $$ C_{g}^{\text{opti}}=1+\frac{f(\varepsilon_{K},\phi_{K})}{\nu} $$

with the function

146 $$ \begin{array}{@{}rcl@{}} f&=&\frac{1}{2}\left[\pi-\arcsin\left( \frac{1}{1.38 \cos^{2}\varepsilon_{K}}\right)-2\phi_k\right] \\ &+&1.38 \cdot (1-p_{\text{opti}}(\varepsilon_{K})). \end{array} $$

Finally, we can determine the difference between the optimal cost using the continuous or interrupted Grover algorithm by

147 $$ C_{s}^{\text{opti}}-C_{g}^{\text{opti}}=\frac{1.38(1-p_{K})-f(\varepsilon_{K},\phi_{K})}{\nu} $$

Thus, there exist *j* such that *C*_*g*_(*j*) ≤ *C*_*s*_ if $1.38\cos \limits ^{2}\varepsilon _{K}>1$. For $1.38\cos \limits ^{2}\varepsilon _{K}<1$, we achieve a minimal cost at *j* = 0 such that we do not obtain any advantage continuing the Grover algorithm. In general, the maximal possible different scales as $C_{s}(\psi )-C_{g}(\psi )\sim 1/\nu \sim \sqrt {N^{\prime }}$ if we assume that the classical probability to find a target state with the second oracle is given by $\sin \limits ^{2} \nu =|W|/N=1/N^{\prime }$ (see Fig. 3) .

## Footnotes

1 In recent times, due to progress in quantum-inspired algorithms, the domain of algorithms where exponential speed-ups are to be expected has reduced, but many possibilities for classically intractable computations still exist.

2 More generally, we can allow only states for which, under no quantum channel, allow us to determine such *x* with probability better than given by Grover iterations. This setting is a bit more involved, but it should be clear that as long as this probability is very small, whatever we do in the next phase, cannot be much better than starting from scratch.
